# Supplementary figures and images for: Quantitative proteome comparison of human hearts with those of model organisms
Source: PLoS Biol. 2021 Apr 19;19(4):e3001144. doi: 10.1371/journal.pbio.3001144 (PMC8084454; doi:10.1371/journal.pbio.3001144)

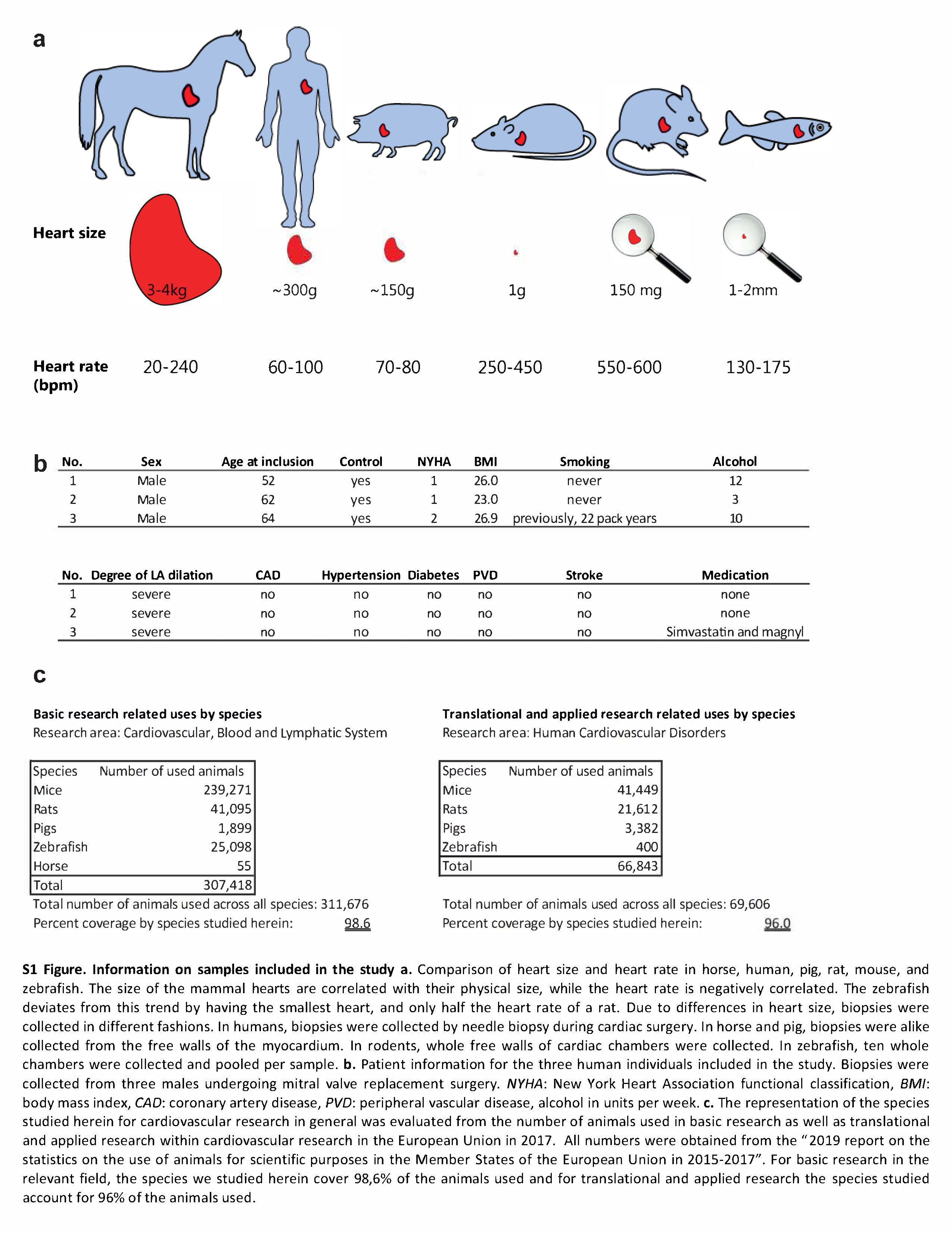

Supplement: S1 Fig — (a) Comparison of heart size and heart rate in horse, human, pig, rat, mouse, and zebrafish. The size of the mammal hearts is correlated with their physical size, while the heart rate is negatively correlated. The zebrafish deviates from this trend by having the smallest heart, and only half the heart rate of a rat. Due to differences in heart size, biopsies were collected in different fashions. In humans, biopsies were collected by needle biopsy during cardiac surgery. In horse and pig, biopsies were alike collected from the free walls of the myocardium. In rodents, whole free walls of cardiac chambers were collected. In zebrafish, 10 whole chambers were collected and pooled per sample. (b) Patient information for the 3 human individuals included in the study. Biopsies were collected from 3 males undergoing mitral valve replacement surgery. (c) The representation of the species studied herein for cardiovascular research in general was evaluated from the number of animals used in basic research as well as translational and applied research within cardiovascular research in the European Union in 2017. All numbers were obtained from the “2019 report on the statistics on the use of animals for scientific purposes in the Member States of the European Union in 2015–2017.” For basic research in the relevant field, the species we studied herein cover 98.6% of the animals used, and for translational and applied research, the species studied account for 96% of the animals used. BMI, body mass index; CAD, coronary artery disease; NYHA, New York Heart Association functional classification; PVD, peripheral vascular disease, alcohol in units per week. (TIF) [file pbio.3001144.s011.tif]

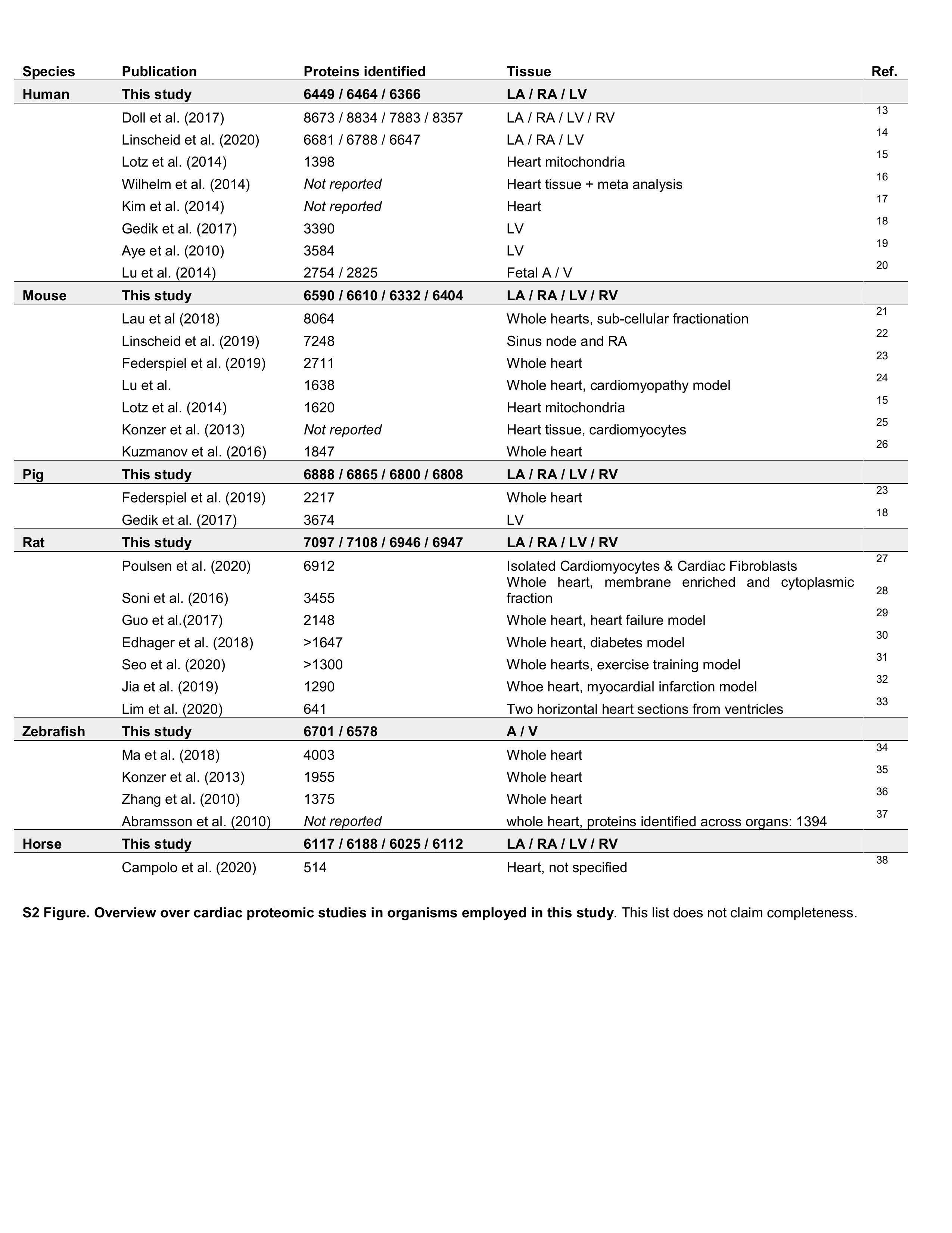

Supplement: S2 Fig — This list does not claim completeness. (TIF) [file pbio.3001144.s012.tif]

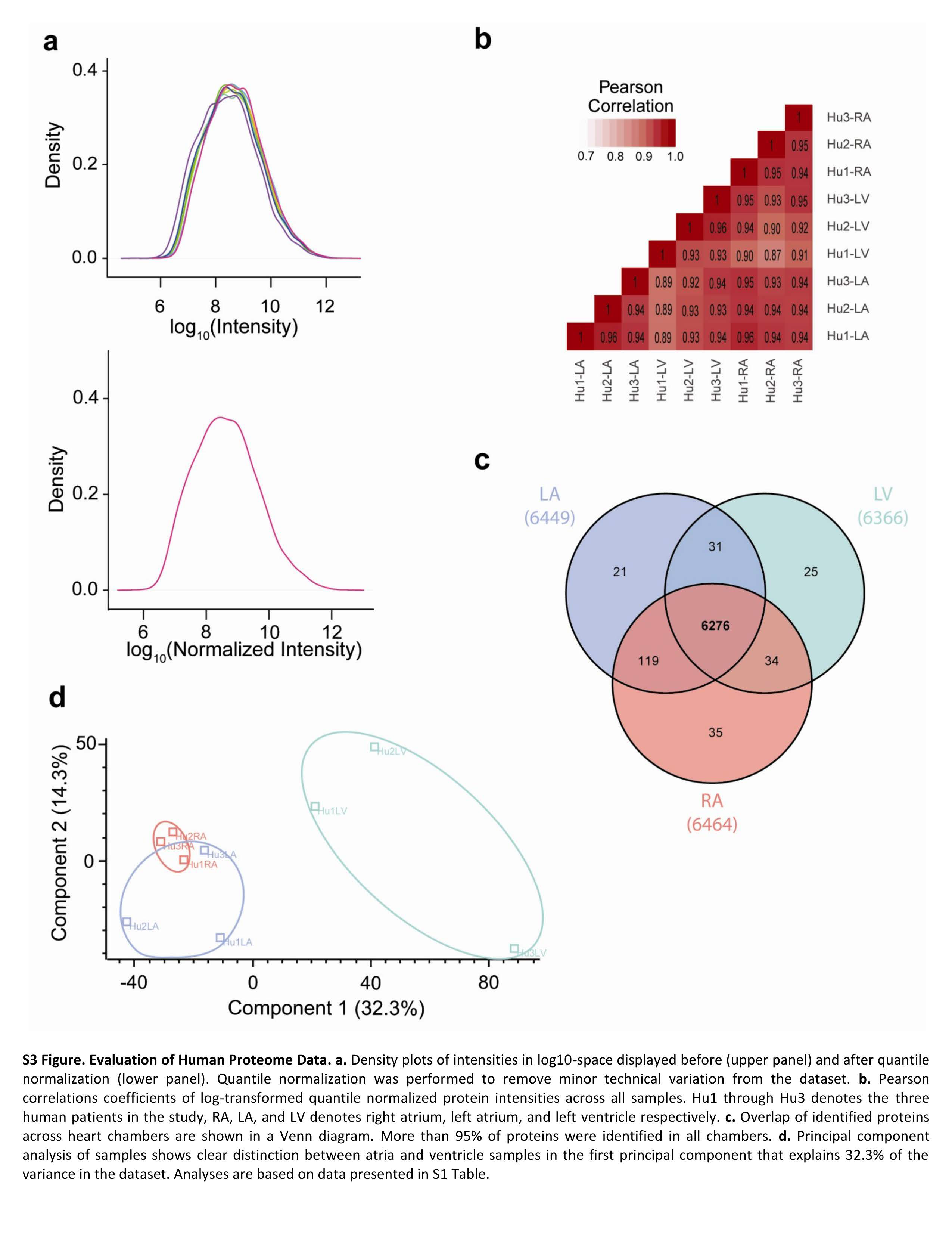

Supplement: S3 Fig — (a) Density plots of intensities in log10-space displayed before (upper panel) and after quantile normalization (lower panel). Quantile normalization was performed to remove minor technical variation from the dataset. (b) Pearson correlations coefficients of log-transformed quantile normalized protein intensities across all samples. Hu1 through Hu3 denote the 3 human patients in the study, and RA, LA, and LV denote right atrium, left atrium, and left ventricle, respectively. (c) Overlap of identified proteins across heart chambers are shown in a Venn diagram. More than 95% of proteins were identified in all chambers. (d) PCA of samples shows clear distinction between atria and ventricle samples in the first principal component that explains 32.3% of the variance in the dataset. Analyses are based on data presented in S1 Table. LA, left atria; LV, left ventricle; PCA, principal component analysis; RA, right atria. (TIF) [file pbio.3001144.s013.tif]

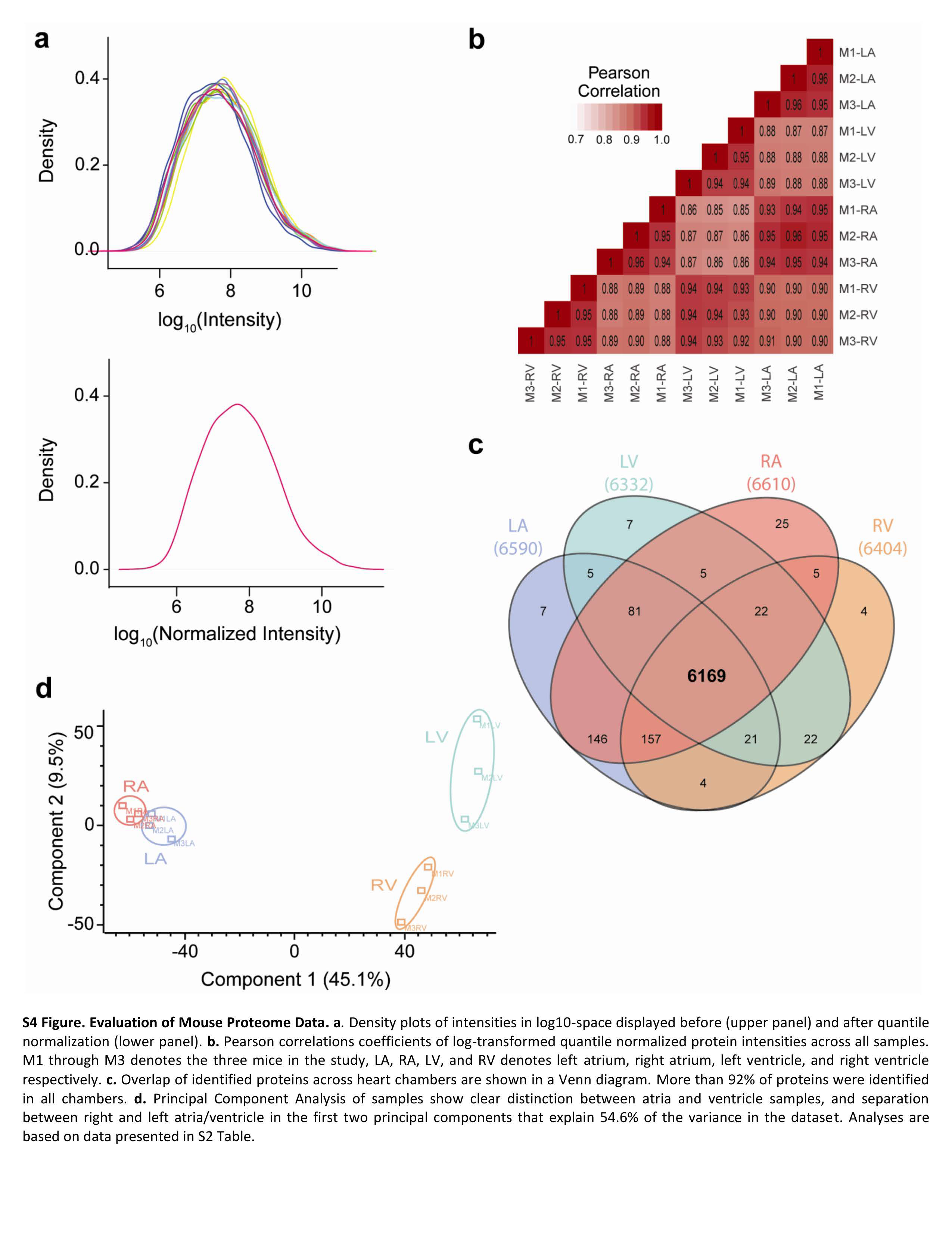

Supplement: S4 Fig — (a) Density plots of intensities in log10-space displayed before (upper panel) and after quantile normalization (lower panel). (b) Pearson correlation coefficients of log-transformed quantile normalized protein intensities across all samples. M1 through M3 denote the 3 mice in the study, and LA, RA, LV, and RV denote left atrium, right atrium, left ventricle, and right ventricle, respectively. (c) Overlap of identified proteins across heart chambers are shown in a Venn diagram. More than 92% of proteins were identified in all chambers. (d) PCA of samples shows clear distinction between atria and ventricle samples, and separation between right and left atria/ventricle in the first 2 principal components that explain 54.6% of the variance in the dataset. Analyses are based on data presented in S2 Table. LA, left atria; LV, left ventricle; PCA, principal component analysis; RA, right atria; RV, right ventricle. (TIF) [file pbio.3001144.s014.tif]

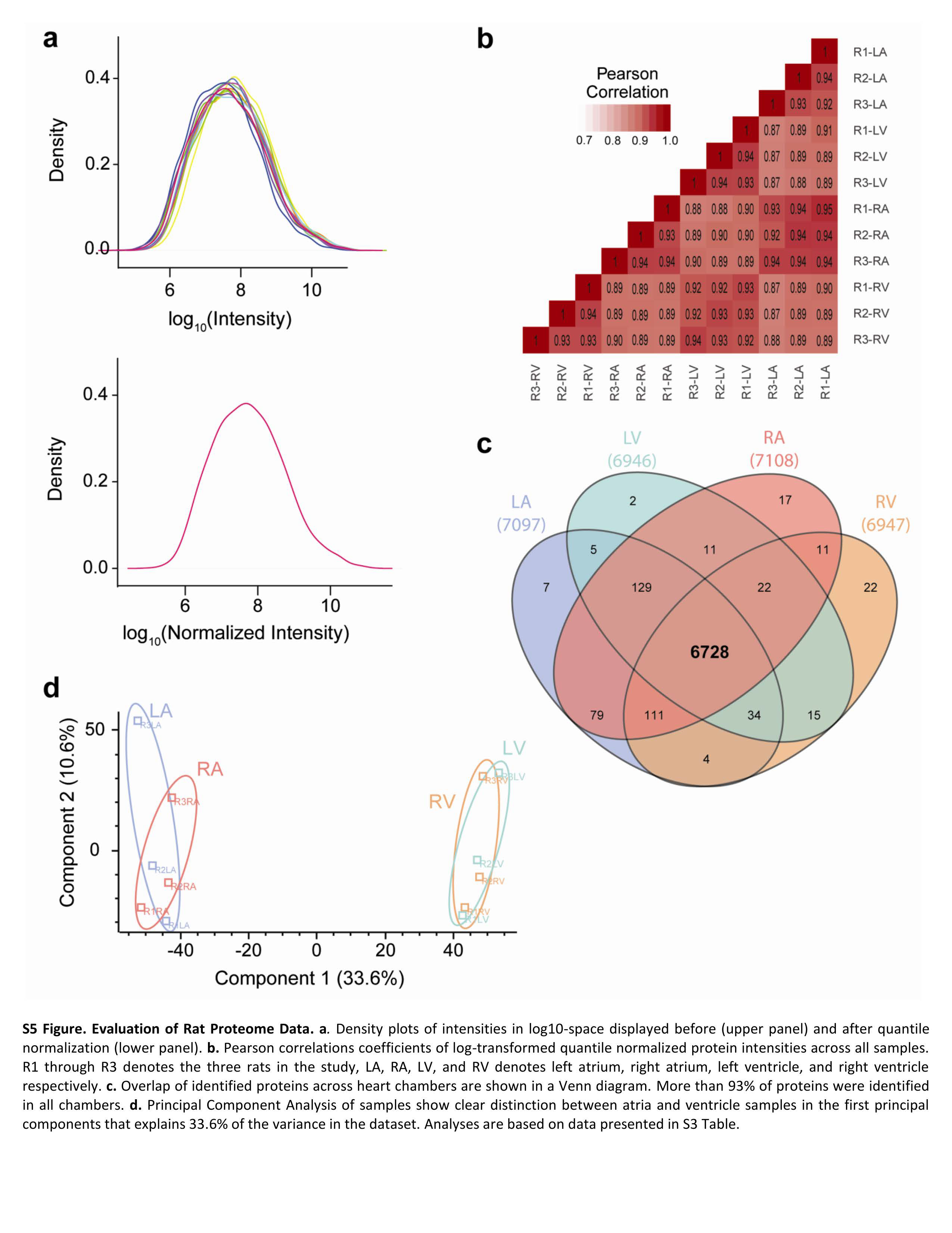

Supplement: S5 Fig — (a) Density plots of intensities in log10-space displayed before (upper panel) and after quantile normalization (lower panel). (b) Pearson correlation coefficients of log-transformed quantile normalized protein intensities across all samples. R1 through R3 denote the 3 rats in the study, and LA, RA, LV, and RV denote left atrium, right atrium, left ventricle, and right ventricle, respectively. (c) Overlap of identified proteins across heart chambers are shown in a Venn diagram. More than 93% of proteins were identified in all chambers. (d) PCA of samples shows clear distinction between atria and ventricle samples in the first principal components that explains 33.6% of the variance in the dataset. Analyses are based on data presented in S3 Table. LA, left atria; LV, left ventricle; PCA, principal component analysis; RA, right atria; RV, right ventricle. (TIF) [file pbio.3001144.s015.tif]

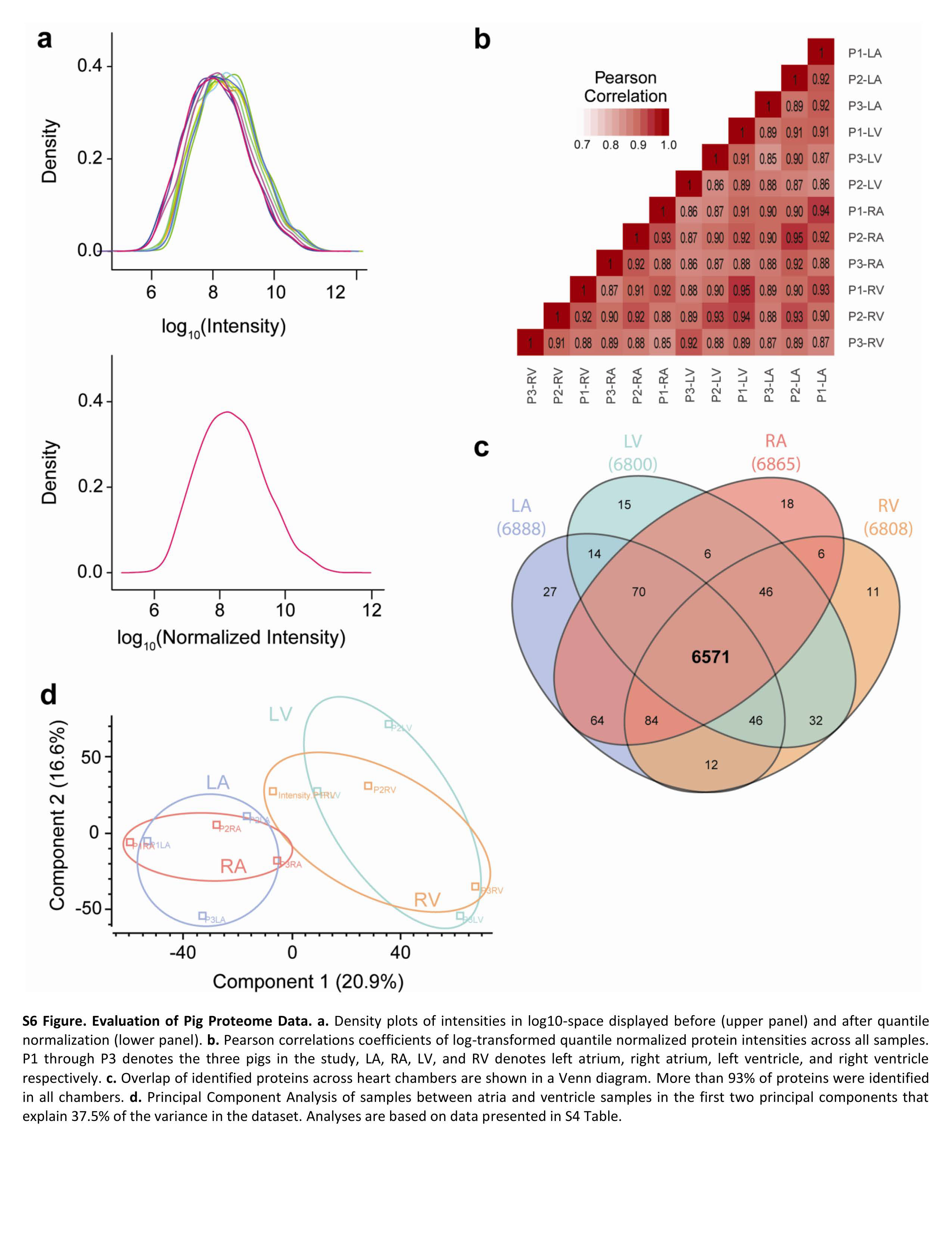

Supplement: S6 Fig — (a) Density plots of intensities in log10-space displayed before (upper panel) and after quantile normalization (lower panel). (b) Pearson correlation coefficients of log-transformed quantile normalized protein intensities across all samples. P1 through P3 denote the 3 pigs in the study, and LA, RA, LV, and RV denote left atrium, right atrium, left ventricle, and right ventricle, respectively. (c) Overlap of identified proteins across heart chambers are shown in a Venn diagram. More than 93% of proteins were identified in all chambers. (d) PCA of samples shows distinction between atria and ventricle samples in the first 2 principal components that explain 37.5% of the variance in the dataset. Analyses are based on data presented in S4 Table. LA, left atria; LV, left ventricle; PCA, principal component analysis; RA, right atria; RV, right ventricle. (TIF) [file pbio.3001144.s016.tif]

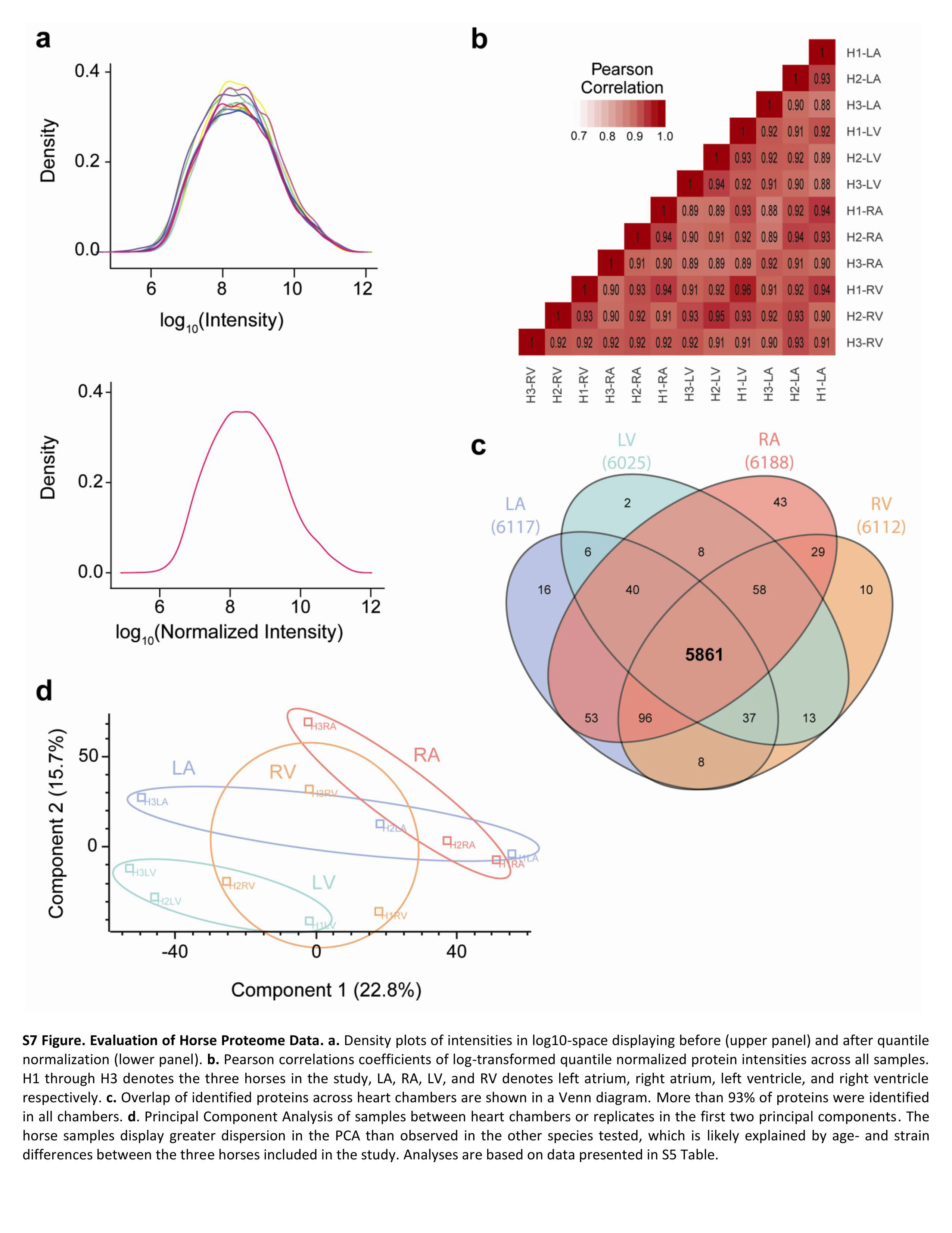

Supplement: S7 Fig — (a) Density plots of intensities in log10-space displaying before (upper panel) and after quantile normalization (lower panel). (b) Pearson correlation coefficients of log-transformed quantile normalized protein intensities across all samples. H1 through H3 denote the 3 horses in the study, and LA, RA, LV, and RV denote left atrium, right atrium, left ventricle, and right ventricle, respectively. (c) Overlap of identified proteins across heart chambers are shown in a Venn diagram. More than 93% of proteins were identified in all chambers. (d) PCA of samples showing heart chambers or replicates along the first 2 principal components. The horse samples display greater dispersion in the PCA than observed in the other species tested, which is likely explained by age and strain differences between the 3 horses included in the study. Analyses are based on data presented in S5 Table. LA, left atria; LV, left ventricle; PCA, principal component analysis; RA, right atria; RV, right ventricle. (TIF) [file pbio.3001144.s017.tif]

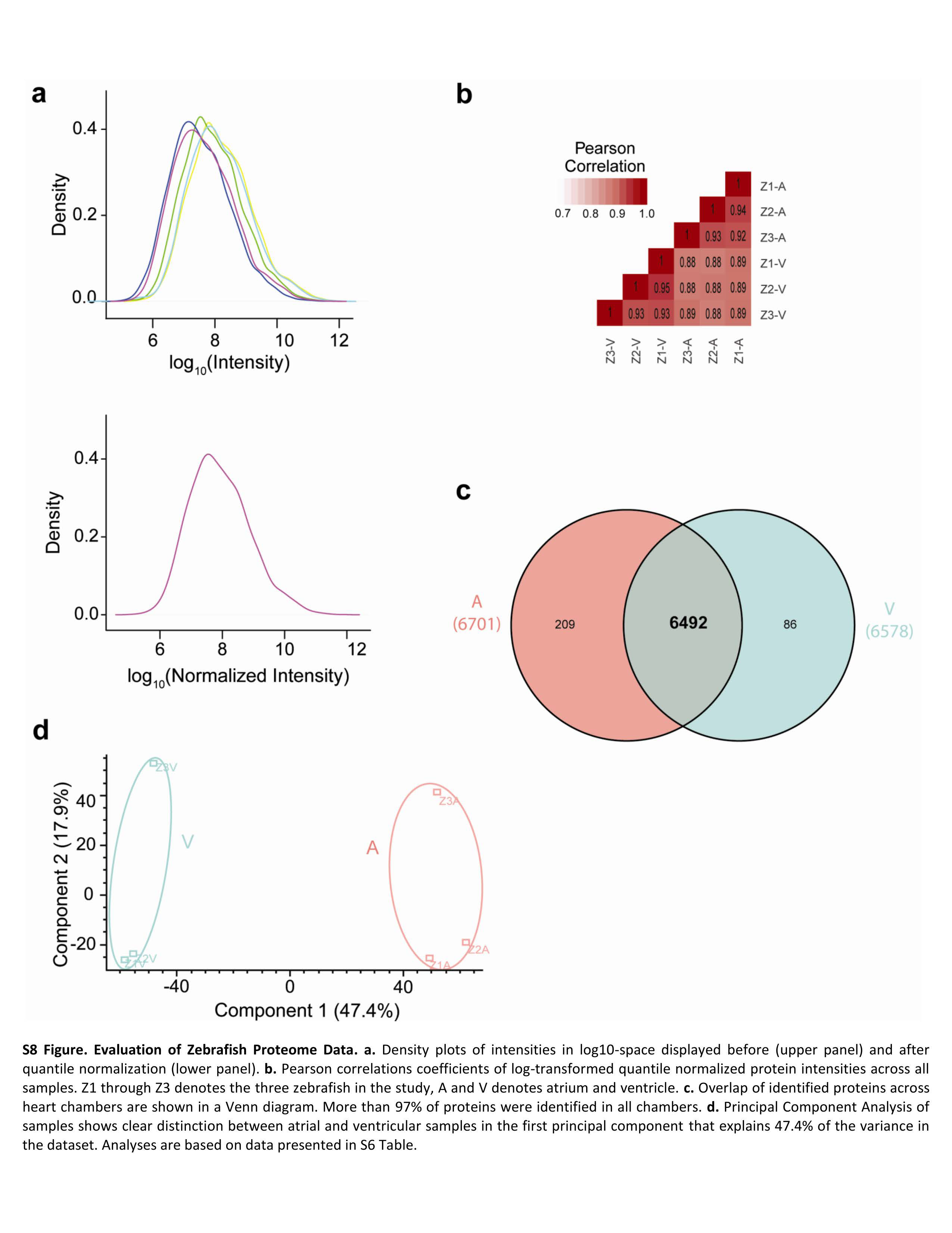

Supplement: S8 Fig — (a) Density plots of intensities in log10-space displayed before (upper panel) and after quantile normalization (lower panel). (b) Pearson correlation coefficients of log-transformed quantile normalized protein intensities across all samples. Z1 through Z3 denote the 3 zebrafish in the study, and A and V denote atrium and ventricle. (c) Overlap of identified proteins across heart chambers are shown in a Venn diagram. More than 97% of proteins were identified in all chambers. (d) PCA of samples shows clear distinction between atrial and ventricular samples in the first principal component that explains 47.4% of the variance in the dataset. Analyses are based on data presented in S6 Table. PCA, principal component analysis. (TIF) [file pbio.3001144.s018.tif]

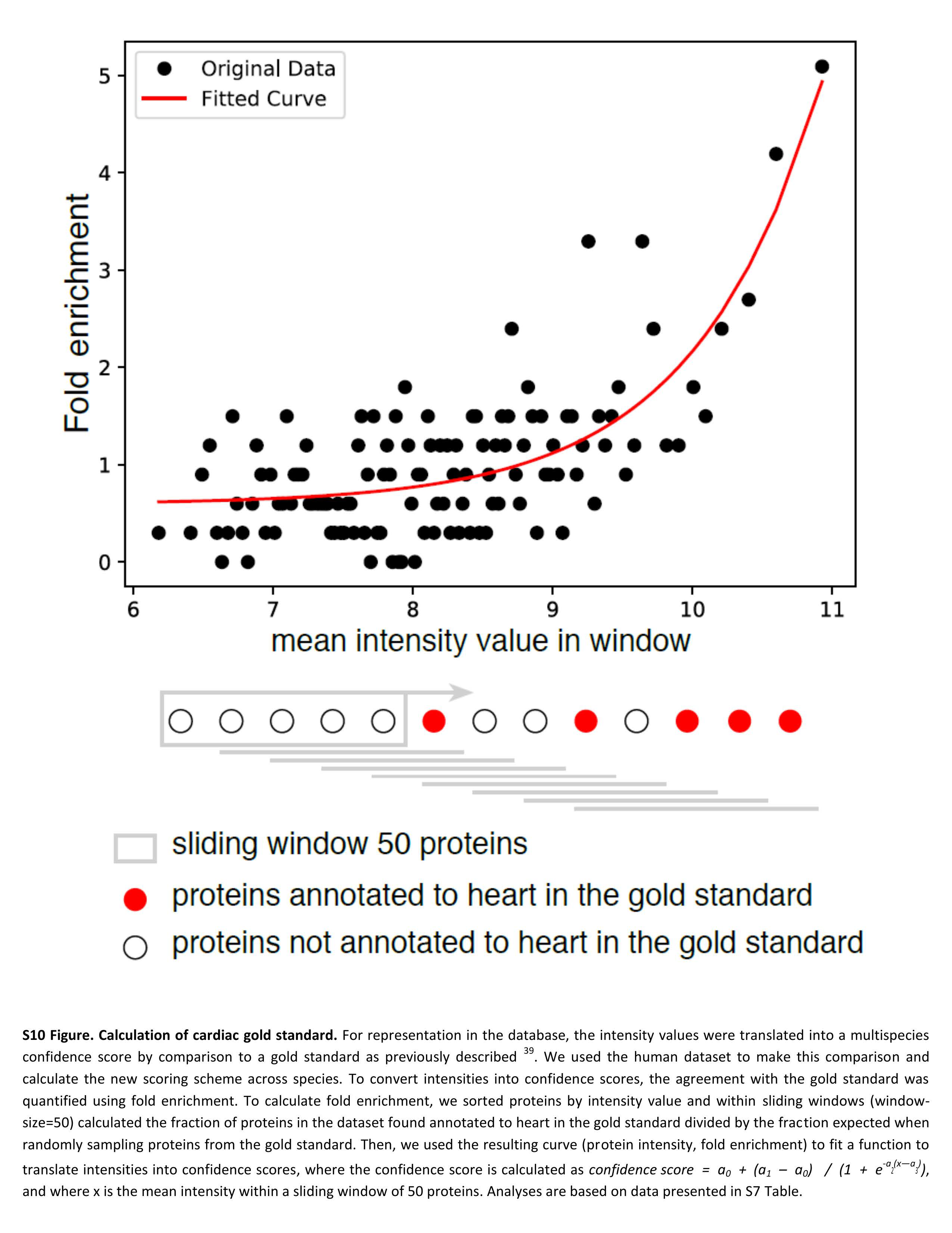

Supplement: S10 Fig — For representation in the database, the intensity values were translated into a multispecies confidence score by comparison to a gold standard as previously described [21]. We used the human dataset to make this comparison and calculate the new scoring scheme across species. To convert intensities into confidence scores, the agreement with the gold standard was quantified using fold enrichment. To calculate fold enrichment, we sorted proteins by intensity value and within sliding windows (window size = 50) calculated the fraction of proteins in the dataset found annotated to heart in the gold standard divided by the fraction expected when randomly sampling proteins from the gold standard. Then, we used the resulting curve (protein intensity, fold enrichment) to fit a function to translate intensities into confidence scores: confidencescore=a0+(a1–a0)/(1+e−a2(x–a3)), where x is the mean intensity within a sliding window of 50 proteins. Analyses are based on data presented in S7 Table. (TIF) [file pbio.3001144.s020.tif]

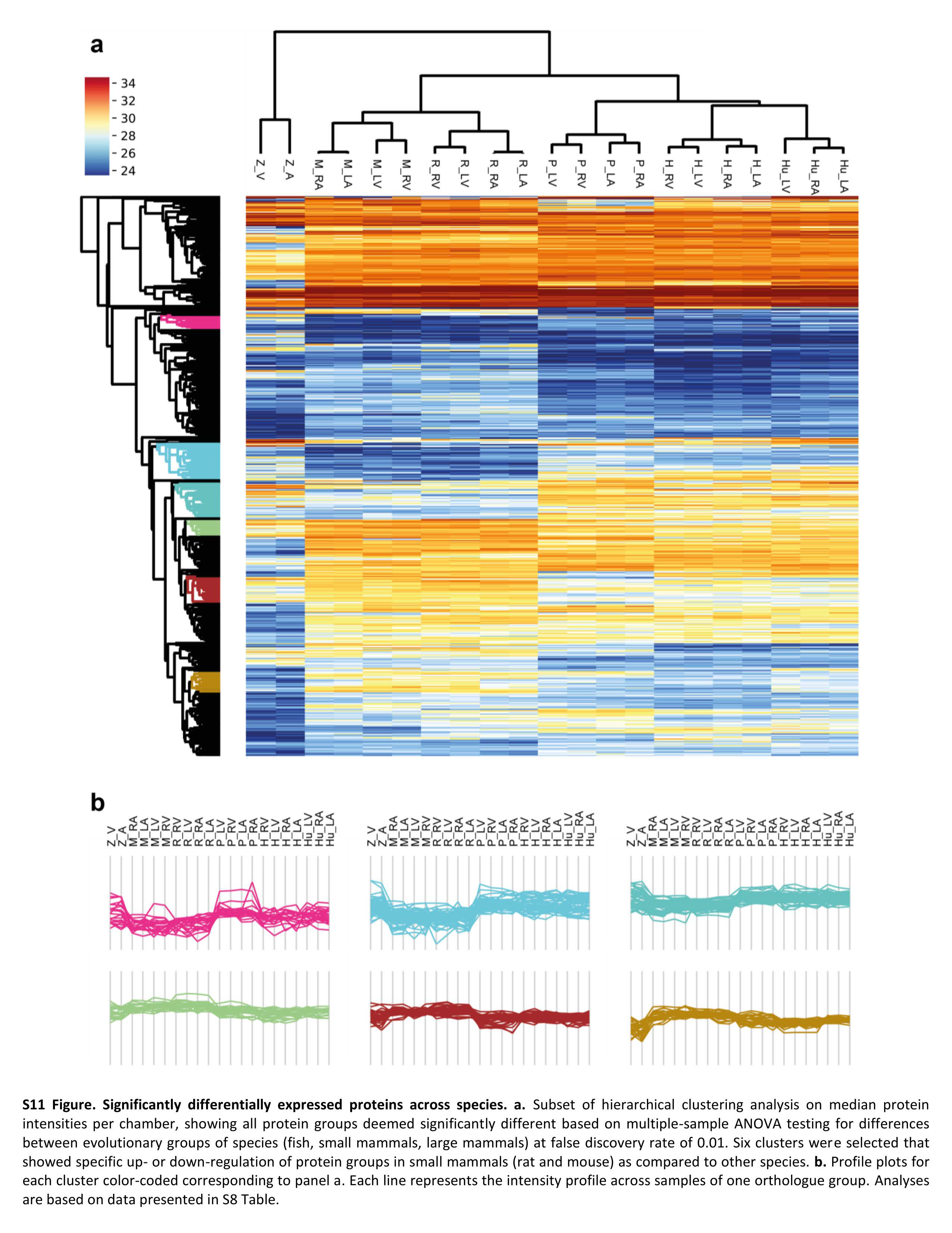

Supplement: S11 Fig — (a) Subset of hierarchical clustering analysis on median protein intensities per chamber, showing all protein groups deemed significantly different based on multiple-sample ANOVA testing for differences between evolutionary groups of species (fish, small mammals, and large mammals) at false discovery rate of 0.01. Six clusters were selected that showed specific up- or down-regulation of protein groups in small mammals (rat and mouse) as compared to other species. (b) Profile plots for each cluster color-coded corresponding to panel a. Each line represents the intensity profile across samples of 1 orthologue group. Analyses are based on data presented in S8 Table. (TIF) [file pbio.3001144.s021.tif]

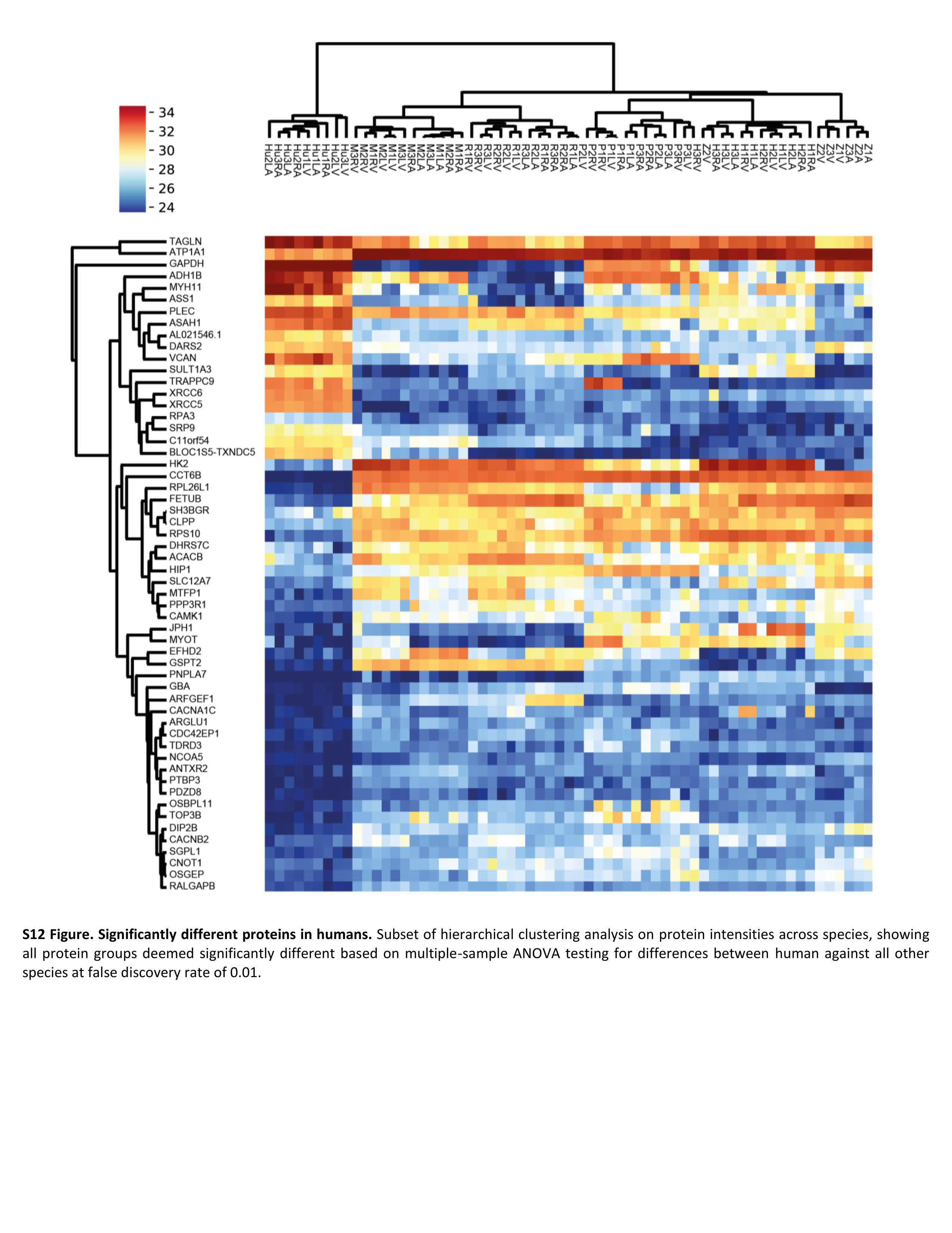

Supplement: S12 Fig — Subset of hierarchical clustering analysis on protein intensities across species, showing all protein groups deemed significantly different based on multiple-sample ANOVA testing for differences between human against all other species at false discovery rate of 0.01. (TIF) [file pbio.3001144.s022.tif]

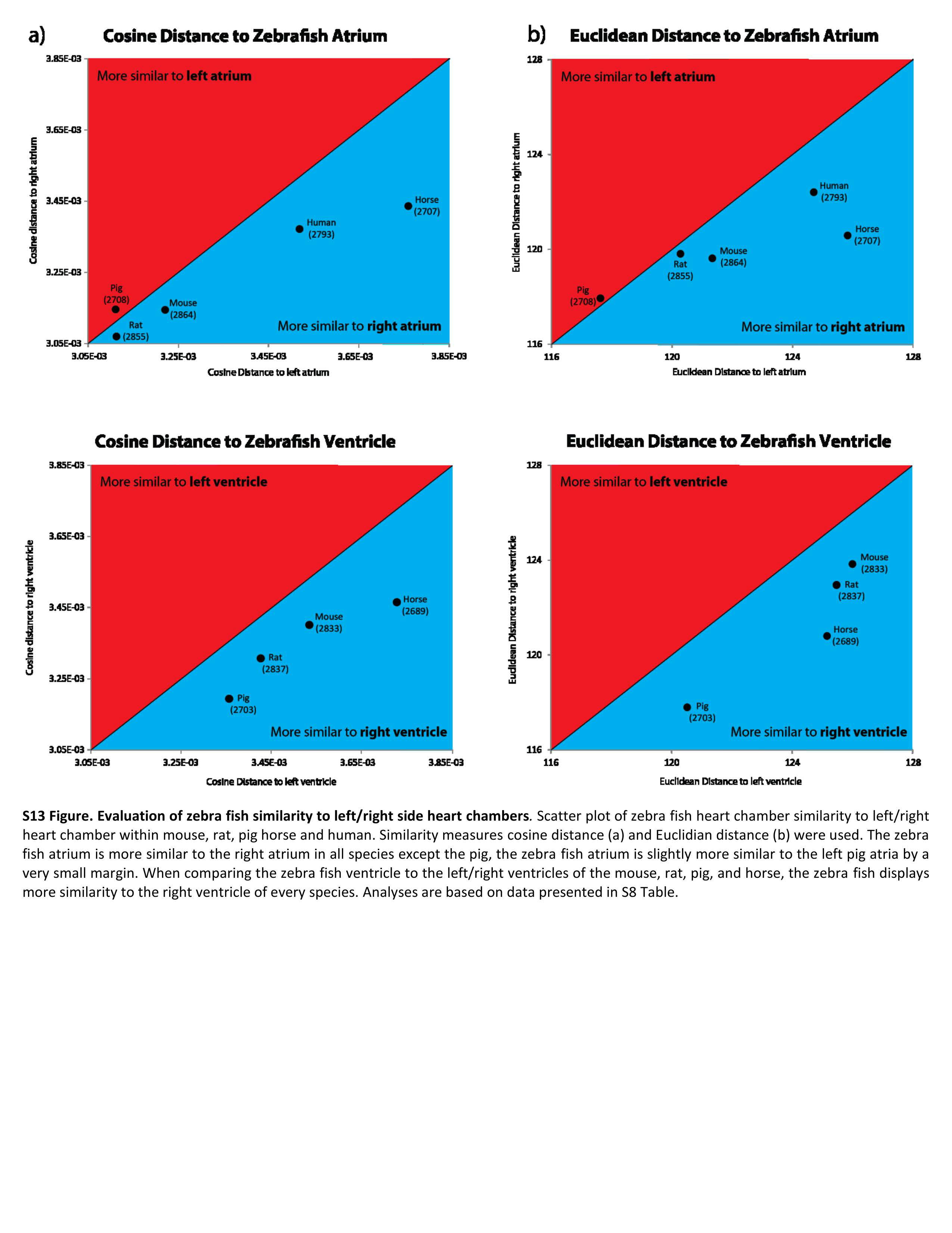

Supplement: S13 Fig — Scatter plot of zebrafish heart chamber similarity to left/right heart chamber within mouse, rat, pig, horse, and human. Similarity measures cosine distance (a) and Euclidean distance (b) were used. The zebrafish atrium is more similar to the right atrium in all species except the pig; the zebrafish atrium is slightly more similar to the left pig atria by a very small margin. When comparing the zebrafish ventricle to the left/right ventricles of the mouse, rat, pig, and horse, the zebrafish displays more similarity to the right ventricle of every species. Analyses are based on data presented in S8 Table. (TIF) [file pbio.3001144.s023.tif]

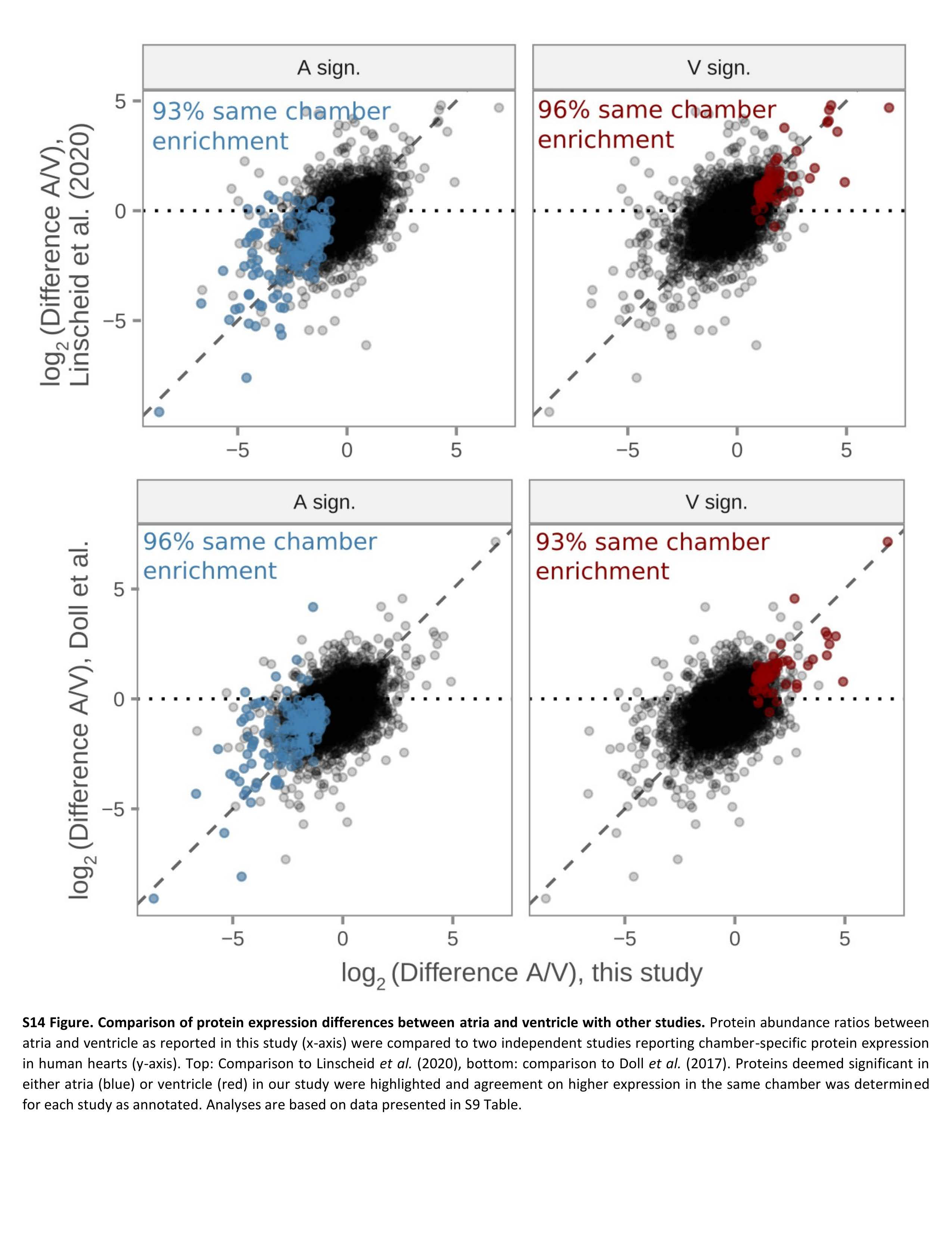

Supplement: S14 Fig — Protein abundance ratios between atria and ventricle as reported in this study (x-axis) were compared to 2 independent studies reporting chamber-specific protein expression in human hearts (y-axis). Top: comparison to Linscheid et al. (2020) [14]. Bottom: comparison to Doll et al. (2017) [39]. Proteins deemed significant in either atria (blue) or ventricle (red) in our study were highlighted, and agreement on higher expression in the same chamber was determined for each study as annotated. Analyses are based on data presented in S9 Table. (TIF) [file pbio.3001144.s024.tif]

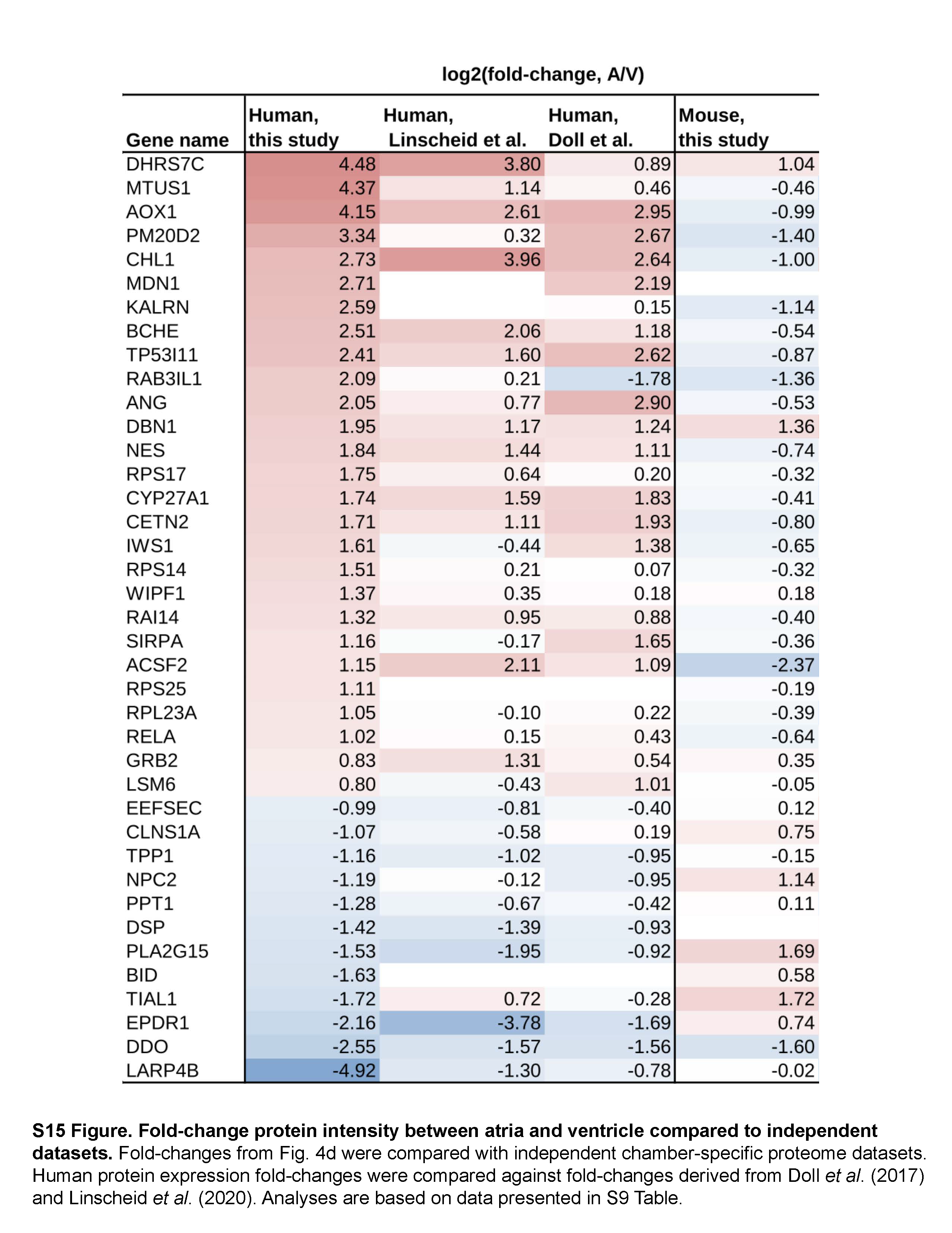

Supplement: S15 Fig — Fold changes from Fig 4D were compared with independent chamber-specific proteome datasets. Human protein expression fold changes were compared against fold changes derived from Doll et al. (2017) [39] and Linscheid et al. (2020) [14]. Analyses are based on data presented in S9 Table. (TIFF) [file pbio.3001144.s025.tiff]

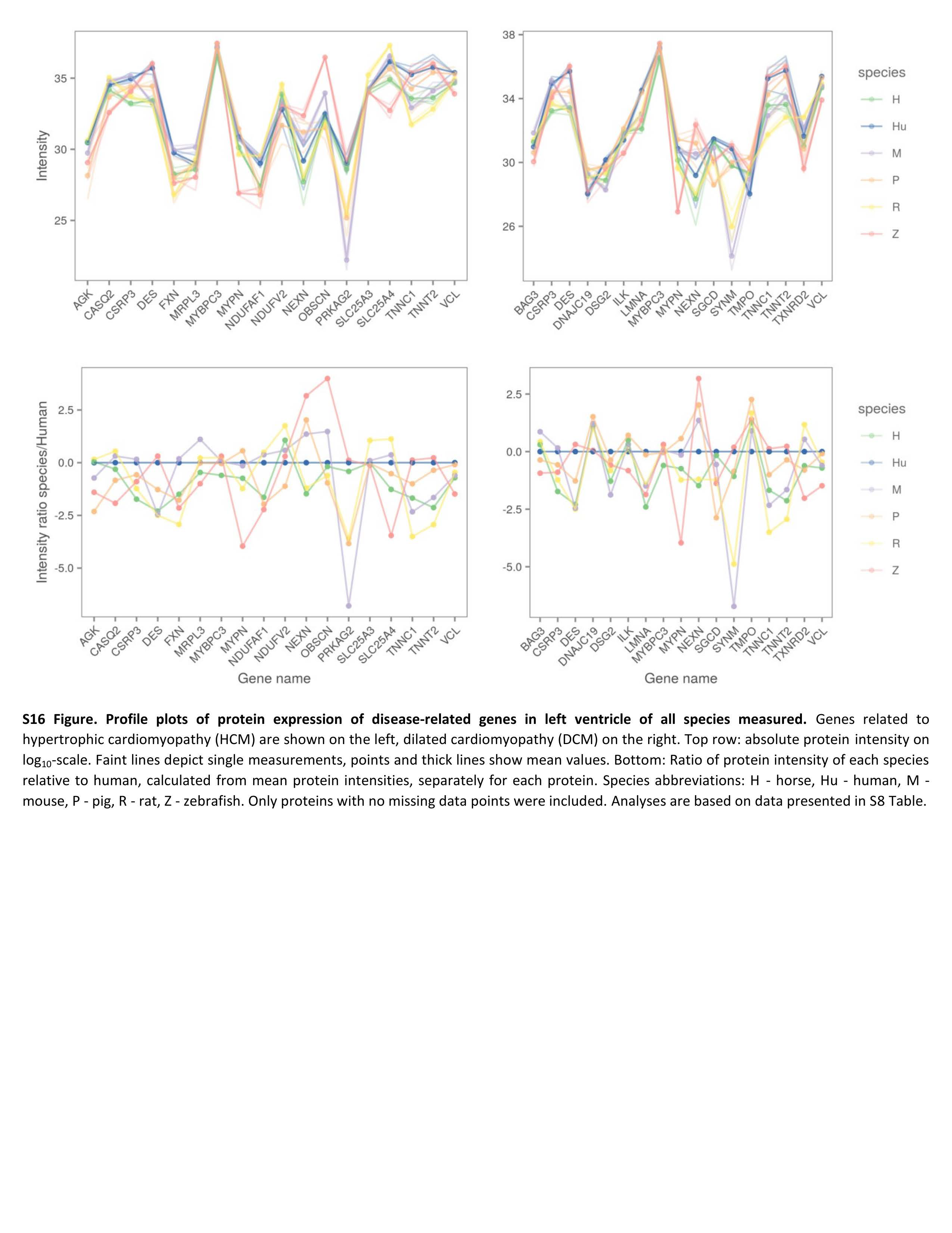

Supplement: S16 Fig — Genes related to HCM are shown on the left and DCM on the right. Top row: absolute protein intensity on log10 scale. Faint lines depict single measurements, and points and thick lines show mean values. Bottom: ratio of protein intensity of each species relative to human, calculated from mean protein intensities, separately for each protein. Species abbreviations: H, horse; Hu, human; M, mouse; P, pig; R, rat; Z, zebrafish. Only proteins with no missing data points were included. Analyses are based on data presented in S8 Table. DCM, dilated cardiomyopathy; HCM, hypertrophic cardiomyopathy. (TIF) [file pbio.3001144.s026.tif]
