# Supplementary material for: Quantitative proteome comparison of human hearts with those of model organisms
Source: PLoS Biol. 2021 Apr 19;19(4):e3001144. doi: 10.1371/journal.pbio.3001144 (PMC8084454; doi:10.1371/journal.pbio.3001144)

Point-by-point Response to Reviewers on MS ID#: PBIOLOGY-D-20-02615R1.

All replies are highlighted in blue font.

Comments from Reviewer 1:

I was happy to review this manuscript by the Lundby group. The authors present a very comprehensive and detailed proteomic assessment of a number of cardiac tissues, representing the largest single proteomic study of its kind. They are to be commended on the depth and quality of this work. The repository that they have put together represents a very valuable addition to the field. I am particularly pleased to see the searchable online database: atlas.cardiacproteomics.com. [I did not search or query their associated PRIDE repository].

I had absolutely no concerns of the methods, protocols, interpretation, or discussion. In fact, I was not able to find even any spelling mistakes or errors! This is very surprising, but very refreshing!! I have not seen such a polished manuscript in over a decade and the authors have clearly invested a considerable amount of energy to get this paper into such top quality. As such, I am very happy to recommend publication of this manuscript.

We are very grateful to the reviewer for the time he/she has invested in reviewing our work, and not least for the exceedingly positive evaluation. It is greatly appreciated that the reviewer praises the quality of our work, especially given how rare this is in a revision process.

Very minor queries at the scientific level.

I am not sure that Eggnogg is the most current approach for homology/paralogue searches. It is certainly acceptable, but I believe it is a little outdated perhaps. OrthoDb or orthognc may be a more current approach.

The reviewer is indeed correct that there are several strategies that can be applied for orthology assignment. For practical reasons (such as the latter implementation of a 'gold standard evaluation') we chose eggNOG for this study. eggNOG has shown to perform well in comparison with other resources (published in 'Standardised Benchmarking in the Quest for Orthologs' by Altenhoff et al in Nature Methods 2016) and is updated and improved periodically. The more important point is that for identification of orthologs over short evolutionary distances, as done herein, methods will agree on the orthology assignments for the vast majority of genes.

We have added the following to the Supplementary material citing the paper mentioned in the response:

"EggNOG has been shown to perform well in comparison with other resources, and it is being improved and updated periodically."

Fig 3C. What is meant by 'same regulation'? Would this not represent more of a similar "directionality". The word regulation has perhaps a different connotation in this setting.

We appreciate the constructive suggestion from the reviewer, who is indeed correct that the phrasing in panel C of figure 4 is not optimal. We have modified the figure legend such that it now reads “Pie charts illustrate the percentage of proteins showing same direction of regulations (green), higher abundance in atria in other species in contrast to mouse (orange) and higher abundance in ventricle in other species contrary to finding in mouse (blue).”. Similarly, we have changed the legend in Figure 4C from ‘same regulation’ to ‘regulation in same direction”.

Supplemental data figure S6D - what is the potential reasoning for the significant overlap of the PCA plots in the horse? There is considerable overlap /too much dispersion, particularly between the RA/LA but seems to not separate from the RV. This is contrast to all of the other tissues examined which showed very nice correlations (clear separations). Also in that the pearson correlations look very tight in panel b.

The reviewer is indeed correct that all other species had better separation across cardiac chambers in the PCA than the horse did. When looking at the PCA plots it is evident that the best chamber separation is achieved from inbred mice and zebrafish closely followed by rats. Data dispersion increases when analysing pig and human heart samples. This is to be expected due to the greater biological variation between the individuals. For the horse, there are two factors contributing to making this the animal with the greatest data dispersion: one is the massive size of a horse heart and the associated difficulty in collecting biopsies from the exact same location. The more important difference is though, the differences between the horses included in the study. For the human individuals we minimized variation by including male, caucasian individuals aged 52-64 years. We obtained hearts from horses donated to the University. These horses were all mares, but one was an eight-year-old tinker and the two others were 16-18 years old small mixed breeds. Indeed you observe that one horse (H3) differs from the two others (H1 and H2). The most likely explanation for the greater variation in the horse data is due to this difference. In order for the manuscript to better reflect this, we have adjusted the legend to supplementary figure 6d, such that it now reads:

“Principal Component Analysis of samples between heart chambers or replicates in the first two principal components. The horse samples display greater dispersion in the PCA than observed in the other species tested, which is likely explained by age- and strain differences between the three horses included in the study.”

Overall I found this to be an outstanding publication and look forward to its acceptance.

Thank you so much!

Comments from Reviewer 2:

Review of PBIOLOGY-D-20-02615R1 "Quantitative Proteome Resource across Hearts from Humans and Model Organisms" by Linscheid et al, submitted to PLOS Biology

Overview

Lundby and colleagues report an in depth, qualitative, and regiospecific mass spectrometry-based analysis of the cardiac proteomes of human in comparison to diverse animal models (pig, horse, rat, mouse and zebrafish). The breadth of coverage (~7,000 proteins) is commendable. Their subsequent comparative analysis reveals intriguing differences in protein expression between species (altered chamber specificity) and heart regions (left vs right ventricle vs atria), motivating possible caveats in using certain models to assess human disease, including leads associated with cardiac pathology that display pronounced model specific expression patterns, as well as suggesting candidates for future functional validation experiments. But in its current form, the study offers only limited descriptive value, lacks conceptual novelty, and falls short in terms of illuminating fundamental differences in biochemical function or pathogenesis, or cardiomyocyte specific expression.

Specific comments

Conceptual novelty - Recognizing the limitations of animal models of human disease is important, but it's not made clear how this work significantly advances understanding of the (human) cardiac proteome, which has been reported extensively on previously, as has that of mouse and rat. Hence, while this is a tour de force effort, that provides impressive coverage of the cardiac proteome across diverse organisms, it's not sufficiently clear how pathophysiologists will change their use of models based on these findings. This is compounded by the fact that the biological variance within (versus between) a species is not reported, which makes comparison across models potentially less meaningful.

The authors are encouraged to leverage their data sets and provide more compelling examples of mechanistic insights they have gleaned, for a specific model species or else human, as well as some form of independent validation experiments to verify certain key findings. The authors could better illustrate the functional relevance of the differences they observe, at least for a few select targets, as a proof of concept to more clearly demonstrate the novelty, mechanistic value and potential impact of the resource they have generated.

We would like to thank the reviewer for his/her efforts in evaluating our work outlining the cardiac proteomes across species. It is our impression that some of the data available wasn't recognized by the reviewer and may have contributed to the reduced enthusiasm. The reviewer comments that the biological variance within species is not reported. This is actually not true, as we do have measurements from three biological replicates from each chamber for each

species. As also recognized by the third reviewer the data that we present herein not only has more protein coverage, but in addition also compares the different cardiac chambers, and as such the information contained in this resource surpasses previous published datasets on the subject. Please see below a table now included as a Supplementary Figure 2, which illustrates that the data contained herein is indeed expanding on our knowledge of cardiac proteomes – also for the commonly used model organisms such as mouse and rat. We agree with the reviewer that obtaining specific mechanistic insights is of tremendous value. However, such efforts are beyond the scope of this single study.

New Supplementary Figure 2:

Species	Publication	Proteins identified	Tissue
**Human**	**This study**	**6449/6464/6366**	**LA/RA/LV**
	Doll et al. (2017)	8673/8834/7883/8357	LA/RA/LV/RV
	Linscheid et al. (2020)	6681/6788/6647	LA/RA/LV
	Lotz et al. (2014)	1398	Heart mitochondria
	Wilhelm et al. (2014)	*Not reported*	Heart tissue + meta analysis
	Kim et al. (2014)	*Not reported*	Heart
	Gedik et al. (2017)	3390	LV
	Aye et al. (2010)	3584	LV
	Lu et al. (2014)	2754/2825	Fetal A/V
**Mouse**	**This study**	**6590/6610/6332/6404**	**LA/RA/LV/RV**
	Lau et al (2018)	8064	Whole hearts, sub-cellular fractionation
	Linscheid et al. (2019)	7248	Sinus node and RA
	Federspiel et al. (2019)	2711	Whole heart
	Lu et al.	1638	Whole heart, cardiomyopathy model
	Lotz et al. (2014)	1620	Heart mitochondria
	Konzer et al. (2013)	*Not reported*	Heart tissue, cardiomyocytes
	Kuzmanov et al. (2016)	1847	Whole heart
**Pig**	**This study**	**6888/6865/6800/6808**	**LA/RA/LV/RV**
	Federspiel et al. (2019)	2217	Whole heart
	Gedik et al. (2017)	3674	LV
**Rat**	**This study**	**7097/7108/6946/6947**	**LA/RA/LV/RV**
	Poulsen et al. (2020)	6912	Isolated Cardiomyocytes & Cardiac Fibroblasts
	Soni et al. (2016)	3455	Whole heart, membrane enriched and cytoplasmic fraction
	Guo et al.(2017)	2148	Whole heart, heart failure model
	Edhager et al. (2018)	>1647	Whole heart, diabetes model
	Seo et al. (2020)	>1300	Whole hearts, exercise training model
	Jia et al. (2019)	1290	Whoe heart, myocardial infarction model
	Lim et al. (2020)	641	Two horizontal heart sections from ventricles
**Zebrafish**	**This study**	**6701/6578**	**A/V**
	Ma et al. (2018)	4003	Whole heart
	Konzer et al. (2013)	1955	Whole heart
	Zhang et al. (2010)	1375	Whole heart
	Abramsson et al. (2010)	*Not reported*	whole heart, total proteins identified across organs: 1394
**Horse**	**This study**	**6117/6188/6025/6112**	**LA/RA/LV/RV**
	Campolo et al. (2020)	514	Heart, not specified

To exemplify how the data resource presented herein may be utilized to choose preferred model organism for investigation of a particular pathophysiology, we have included an example of how such an analysis can be performed in the revised manuscript. Specifically, we show how to extract information on cardiac protein abundances for proteins encoded by genes involved in hypertrophic cardiomyopathy, and evaluate the protein profiles in the left ventricle across all the species studied. This representation is included as a new supplementary figure S16, as shown below. We have added this figure as “blueprint analysis” to illustrate how researchers can utilize this data resource in their specific research interest. In the figure below, we show how protein

profiles plotted across species for genes related to HCM or DCM. We add on page 8 of the manuscript: “As an example to illustrate how our data can be of use in interpreting or designing studies performed in model organisms, we plotted profiles of protein expression in LV for HCM- and DCM-related genes, depicting how proteins differ in expression between model organisms and human (Figure S16, Sup. Table S8). Protein profiles are generally similar across organisms for both diseases, with some marked departures: For the HCM example, the largest expression differences are seen for MYPN, NEXN and OBSCN expression between zebrafish and human. On the other hand, PRKAG2, SLC25A4, TNNC1 and TNNT2 are similarly abundant between zebrafish and human, while (sometimes strongly) lower abundant in all other mammals. While our data cannot always point to the best model organism for a given disease, it thus points to candidate genes and proteins which may cause differential responses to medication or disease progression between organisms. For a researcher working on specific processes or diseases, such analyses could help explain discrepancies between studies in different model organisms, or prioritize a list of target proteins to attribute these differences to through follow-up studies.”

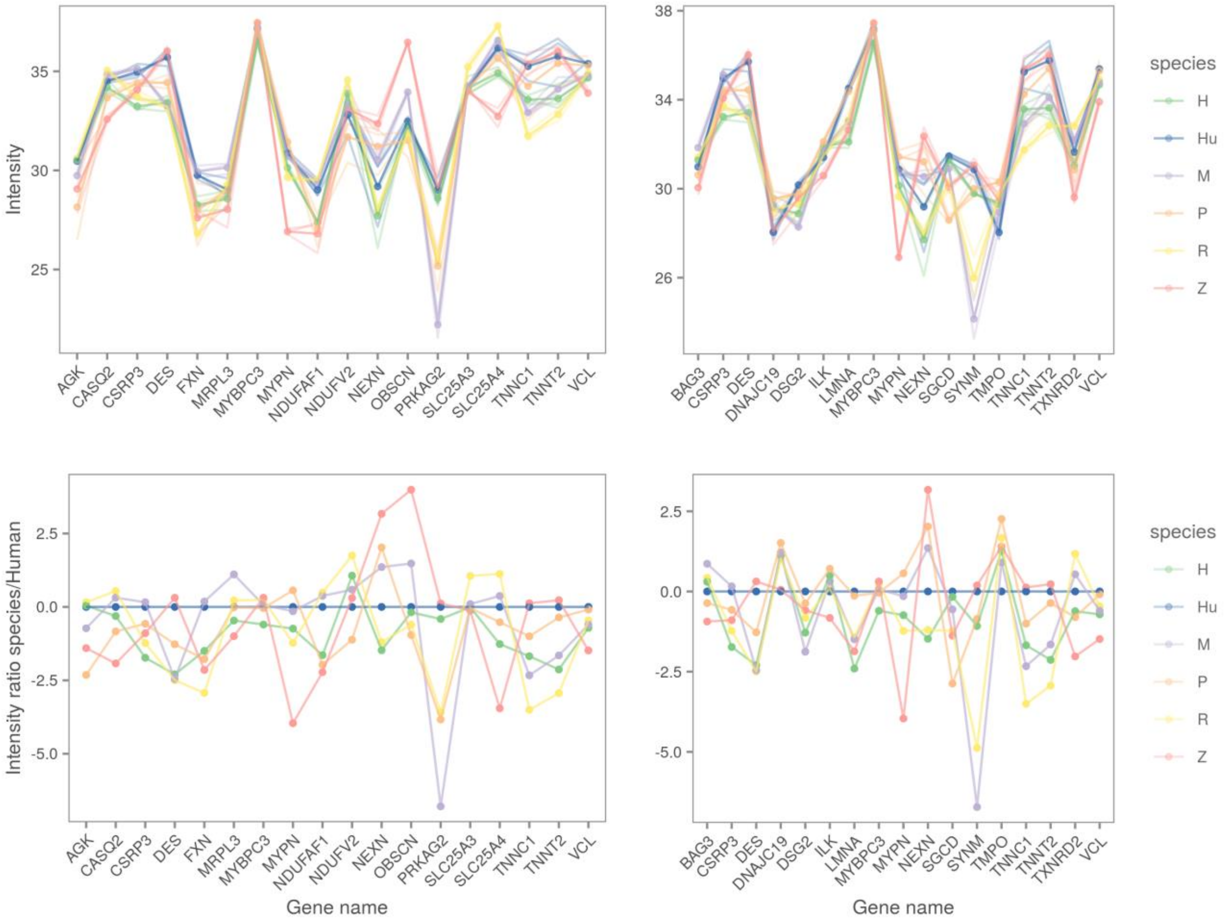

Figure S16: Profile plots of protein expression of disease-related genes in left ventricle of all species measured. Genes related to hypertrophic cardiomyopathy (HCM) are shown on the left, dilated cardiomyopathy (DCM) on the right. Top row: absolute protein intensity on log10-scale. Faint lines depict single measurements, points and thick lines show mean values. Bottom: Ratio of protein intensity of each species relative to human, calculated from mean protein intensities, separately for each protein. Species abbreviations: H - horse, Hu - human, M - mouse, P - pig, R - rat, Z - zebrafish. Only proteins with no missing data points were included.

Comments from Reviewer 3: Edward Lau - note that this reviewer has waived anonymity
Heart diseases are a major cause of morbidity and mortality in the world and multiple animal models are employed in biomedical researchers to identify potential disease mechanisms and translate results to human. The molecular differences between the heart of different model species await further investigations.

To address this, the authors compared the abundance of 7,000 proteins using label free mass spectrometry between human, pig, horse, rat, mouse, and zebrafish. Protein expressions are compared between left ventricles, right ventricles, left atriums and right atriums of the mammalian species, and between the atrium and ventricle for the two-chambered hearts in fish. They found:

* Protein profiles largely clustered by evolutionary lineages as expected

* Nevertheless, important differences in expression of major cardiac proteins (natriuretic peptides, myosin heavy and light chains, etc.) among model animals especially zebrafish

* Unexpected inversion of atrium/ventricle relative protein distributions across species

* Some species are more like human in their expression profiles of some disease-implicated proteins which may be taken into consideration when deciding on disease model.

Overall the looks like a competent study, applying state of the art mass spectrometry to survey of the proteins expressed in different chambers of the heart across five species. The proteomics experiments look to be well executed. There were some previous attempts to compare heart protein expression in different species which should be cited, but the current data set has more protein coverage and also compared different chambers. Some particular strengths of this dataset over previous proteomics comparisons of are the use of freshly collected samples, and resolving orthology in cross-species comparisons. Raw and summarized data are made publicly available for re-analysis, and the authors have created a web app for easy data interactions. I believe it will provide a useful resource for researchers in cardiac biology and human proteomes.

Even though this is primarily a resource study, I think there is an opportunity to provide additional contexts and interpretations for this large dataset. I have the following comments:

Major Comments:

1. There were previous attempts to compare the hearts of different species (e.g., Ref 14 and 15 cited in the manuscript, PMID 24070373, and potentially others). It might be reasonable to

compare them to the current study (in terms of protein and species coverage) and also provide a short analysis of shared proteins and concordance or disagreements between the results.

We would like to thank the reviewer for his efforts evaluating our work. We greatly appreciate the time and effort spent as well as the constructive criticism provided, which we believe have further strengthened the study. Upon suggestion by the reviewer, we have compiled an overview of previously published proteomics datasets of heart tissue. Direct comparisons across these datasets are difficult, due to differences in sample collection, enrichments, preparations and measurements. Nonetheless, we believe that the table compiled presents a useful overview of previously published work on the subject and highlights additional datasets where further information may be acquired. The following has been included in the revised manuscript as a new Supplementary Figure S2:

Species	Publication	Proteins identified	Tissue
**Human**	**This study**	**6449/6464/6366**	**LA/RA/LV**
	Doll et al. (2017)	8673/8834/7883/8357	LA/RA/LV/RV
	Linscheid et al. (2020)	6681/6788/6647	LA/RA/LV
	Lotz et al. (2014)	1398	Heart mitochondria
	Wilhelm et al. (2014)	*Not reported*	Heart tissue + meta analysis
	Kim et al. (2014)	*Not reported*	Heart
	Gedik et al. (2017)	3390	LV
	Aye et al. (2010)	3584	LV
	Lu et al. (2014)	2754/2825	Fetal A/V
**Mouse**	**This study**	**6590/6610/6332/6404**	**LA/RA/LV/RV**
	Lau et al (2018)	8064	Whole hearts, sub-cellular fractionation
	Linscheid et al. (2019)	7248	Sinus node and RA
	Federspiel et al. (2019)	2711	Whole heart
	Lu et al.	1638	Whole heart, cardiomyopathy model
	Lotz et al. (2014)	1620	Heart mitochondria
	Konzer et al. (2013)	*Not reported*	Heart tissue, cardiomyocytes
	Kuzmanov et al. (2016)	1847	Whole heart
**Pig**	**This study**	**6888/6865/6800/6808**	**LA/RA/LV/RV**
	Federspiel et al. (2019)	2217	Whole heart
	Gedik et al. (2017)	3674	LV
**Rat**	**This study**	**7097/7108/6946/6947**	**LA/RA/LV/RV**
	Poulsen et al. (2020)	6912	Isolated Cardiomyocytes & Cardiac Fibroblasts
	Soni et al. (2016)	3455	Whole heart, membrane enriched and cytoplasmic fraction
	Guo et al.(2017)	2148	Whole heart, heart failure model
	Edhager et al. (2018)	>1647	Whole heart, diabetes model
	Seo et al. (2020)	>1300	Whole hearts, exercise training model
	Jia et al. (2019)	1290	Whoe heart, myocardial infarction model
	Lim et al. (2020)	641	Two horizontal heart sections from ventricles
**Zebrafish**	**This study**	**6701/6578**	**A/V**
	Ma et al. (2018)	4003	Whole heart
	Konzer et al. (2013)	1955	Whole heart
	Zhang et al. (2010)	1375	Whole heart
	Abramsson et al. (2010)	*Not reported*	whole heart, total proteins identified across organs: 1394
**Horse**	**This study**	**6117/6188/6025/6112**	**LA/RA/LV/RV**
	Campolo et al. (2020)	514	Heart, not specified

2. How much of the protein expression differences across species might be due to differences in cell type proportion and distribution? In this regard the low loading of mitochondrial proteins across species is a notable results.

This is indeed an interesting question. Unfortunately, the question can't be rigorously addressed from the data we have collected, as the data we have is measured from biopsies containing a mix of all cell types. At present, we do not have record of differences in cell type distributions across the species analyzed here. There is some data available for mouse hearts and some data for human atria, but no datasets that enable us to make such analysis across chambers and across species. As such the question would merit another study with that particular question in focus.

As we recognize the importance of potential cellular differences across chambers as well as across species, we have added the following to the discussion of the manuscript:

"An important next step will be to expand on this resource with information on the cellular composition across the cardiac regions (28) and how these differ across humans and model organisms (42), and from there expanding to evaluate protein abundances per cardiac cell type (43) as technologies improve and allow for it."

3. More generally, there isn't a lot of consideration given to why there are these species specific differences especially with regard to critical cardiac proteins. Which difference may be potentially attributable to differences in heart rate, metabolic needs, turnover, etc.?

The species studied herein are quite diverse. Zebrafish are evolutionary distant ectothermic animals, with a spontaneously beating rate of approx. 150 beats per minute (bpm) at 28 C. Yet, they have been used extensively to investigate cardiovascular development and regeneration. Despite their small size, some of the electrophysiological properties, including the overall shape of the ventricular action potential is closer to that of human hearts than action potentials from murine models. Human, pig and horse cluster together as do mice and rats. Mice and rats have fast heart rates of 310-840 bpm and 330-480, respectively. In accordance with the high beating rate, the cardiac action potentials are very short, and lack a well-defined plateau phase as observed in the larger animals, such as the pig, horse or humans. These differences in electrophysiological properties in general limit the use of mouse and rats as models for human hearts. Pig hearts have been extensively used as models for human hearts, as the hearts are of similar size and the beating rate comparable (70-80 bpm for adult pigs, 60-100 bpm for human adults). Pig heart vasculature is also very similar to humans, and despite minor differences in the distribution of the conduction system, the electrophysiological properties are very similar to those of human hearts. A horse heart weighs up to 4 kg, and the resting heart rate is close to 30 bpm. During exercise, the heart rate can increase up to 240 depending on fitness and breed. The span in heart rate implies significant adaptation of the action potential duration. We agree with the reviewer that it is indeed interesting to aim to explain such cardiac physiological differences between species as those enlisted above at the molecular level. To underscore that the protein abundance measurements we provide are directly correlated with physiological differences, we have expanded on the subject in the revised manuscript, and have added the following to the results section on page 5:

'"To facilitate the faster heart rate, the mouse and rat myocardium need to contract and relax much faster than is the case for the larger mammals. This is reflected in our data as reduced abundances of the slow-twitch myosin heavy chain MYH7 in mouse and rat compared to human, pig and horse. Similarly, with the faster heart rate the mouse and the rat is expected to

have greater Ca^{2+} handling capacities in the sarcoplasmic reticulum than the larger animals. And indeed, we observe greater abundances for the main calcium handling proteins RYR2, ATP2A2 and CASQ2 in mouse and rat compared to human, pig and horse."

4. Some statistics on animal model usage in heart research (e.g., PubMed trends and stats) might be helpful here. Is the horse a particular common model for heart biology as the authors suggest, compared to other animals like rabbits, etc.

There are many different opinions on which are the best model organisms for cardiac research. It is indeed correct that the rabbit is more frequently used than e.g. horse, yet the horse is used by some research environments as the horse spontaneously develop atrial fibrillation (REF DOI: 10.1186/s12872-019-1210-4). To add some statistics for the use of model organisms in cardiac research, we have evaluated the actual use of animals for basic research, as well as in translational and applied research in cardiovascular research. We have done so based on data collected in the European Union in 2017. This data evaluation shows that the species we have included in our study cover 98,6% of the animals used in basic research in the relevant area and 96% of the animals used in translational or applied research in cardiovascular disorders. We have added the following to Supplementary Figure 1 as a new panel C:

Basic research related uses by species
Research area: Cardiovascular, Blood and Lymphatic System

Species	Number of used animals
Mice	239,271
Rats	41,095
Pigs	1,899
Zebrafish	25,098
Horse	55
Total	307,418

Total number of animals used across all species: 311,676
Percent coverage by species studied herein: 98.6

Translational and applied research related uses by species
Research area: Human Cardiovascular Disorders

Species	Number of used animals
Mice	41,449
Rats	21,612
Pigs	3,382
Zebrafish	400
Total	66,843

Total number of animals used across all species: 69,606
Percent coverage by species studied herein: 96.0

New legend for Supplementary Figure 1C: "**c**. The representation of the species studied herein for cardiovascular research in general was evaluated from the number of animals used in basic research as well as translational and applied research within cardiovascular research in the European Union in 2017. All numbers were obtained from the "2019 report on the statistics on the use of animals for scientific purposes in the Member States of the European Union in 2015-2017". For basic research in the relevant field, the species we studied herein cover 98,6% of the animals used and for translational and applied research the species studied account for 96% of the animals used."

We refer to this addition in the manuscript on page 4, where it now says "The quantitative proteomics dataset acquired represents a comprehensive mapping of cardiac protein expression profiles across chambers for human heart and five commonly used model organisms in cardiac research (Supplementary Figure 1C)."

To further emphasize the general use of the species studied herein within cardiovascular research, we have made a number of adjustments to the paper, e.g. the introduction has been adjusted as follows: "Priority should be given to species that are anatomically and pathophysiologically similar to the target disease. In more than half of studies testing regenerative medicine in cardiovascular diseases, the pig was the animal of choice (3). The pig has also successfully served as model for cardiac arrhythmias (4,5)."

5. Mapping proteins across species using an orthology database (EggNOG) is a strength and something not always done thoroughly in previous studies that compare species proteome. With the orthology data, can the authors estimate how much of the inter-species qualitative differences in major cardiac proteins are primarily "genomic" in origin (e.g., diversification after gene duplication etc.) and how much is exclusively "proteomic", e.g., all orthologs present in genome difference in tissue expression preference, etc.?

We are grateful that the reviewer acknowledges the efforts involved in mapping the acquired proteomics datasets across species. It would indeed be interesting to evaluate the contribution to species differences at the genomic, proteomic and for that matter transcriptomic level. From the data we have, we could potentially evaluate how protein abundances across species differ for genes that have 1:1 orthologs, and evaluate how this compares to a similar evaluation of differences in protein abundances across species for proteins encoded by genes with multiple orthologs/paralogs. However, the insights that may be obtained from such an approach will be limited, as there are too many unknown factors between the gene level and the protein level that can affect the protein level differently across species. We have no means to evaluate how transcriptional regulation, translational regulations or other biological processes such as protein degradation differ across species, and alike we cannot assume that they are all conserved across species. Thus, we agree with the reviewer that it would be a very interesting insight to obtain. Yet, with the dataset at hand, we cannot draw a general conclusion on inter-species differences driven at either the genomic or the proteomic level.

6. Model selection is often based on a variety of factors, including availability of reagents, ease of genetic manipulation, animal size for surgical model, costs, etc. It is not immediately clear where protein expression network comes in to play here or what is its relative importance. There is a tendency to gravitate toward common models and in so doing "miss out" on unique adaptations that can be informative. It would be more interesting if the authors could hypothesize based on the data what processes could the zebrafish/horse/etc. be a particularly good model for that we are not paying enough attention to.

That is certainly a good point. And for exactly that reason, we have highlighted that based on the proteomics data, we propose that the zebrafish heart is in particular suited for studying phenotypes of the right side of the mammalian heart and that the pig is expected to be the best model organism for studying ARVC. These conclusions are purely based on our proteomics analyses.

In the manuscript we write on page 3: “we show why structural studies of hypertrophic cardiomyopathy are difficult to perform in zebrafish, and we conclude that the best animal model for arrhythmogenic right ventricular cardiomyopathy is pig”

And on page 7: “We examined which side of the mammalian heart the two-chambered zebrafish heart resembles most with regards to its molecular profile. Our analyses consistently showed greatest similarity between zebrafish heart and the right half of mammalian hearts (Fig. 4B and Supplementary Figure S11). This was the case for atria as well as ventricle. We propose this to reflect the zebrafish circulatory system being a low-pressure system, and hence the function of the heart resembling the right side of mammalian hearts serving the lower-pressure pulmonary system.”

Thus, we have indeed utilized our proteomics dataset to propose suitable model organisms in a new manner.

Which model organism would particularly well represent certain processes strongly depends on the question asked, and cannot be answered globally across thousands of protein profiles. Since analyzing each single possible process in question is beyond the scope of this study, we suggest adding one additional figure as “blueprint analysis” to our study to illustrate how researchers can investigate their specific research questions based on our dataset. In the figure below (new Figure S16), we show how protein profiles plotted across species for genes related to HCM or DCM can be of use to cardiac researchers working on a specific question. We add on page 8 of the manuscript: “As an example to illustrate how our data can be of use in interpreting or designing studies performed in model organisms, we plotted profiles of protein expression in LV for HCM- and DCM-related genes, depicting how proteins differ in expression between model organisms and human (Figure S16, Sup. Table S8). Protein profiles are generally similar across organisms for both diseases, with some marked departures: For the HCM example, the largest expression differences are seen for MYPN, NEXN and OBSCN expression between zebrafish and human. On the other hand, PRKAG2, SLC25A4, TNNC1 and TNNT2 are similarly abundant between zebrafish and human, while (sometimes strongly) lower abundant in all other mammals. While our data cannot always point to the best model organism for a given disease, it thus points to candidate genes and proteins which may cause differential responses to medication or disease progression between organisms. For a researcher working on specific processes or diseases, such analyses could help explain discrepancies between studies in different model organisms, or prioritize a list of target proteins to attribute these differences to through follow-up studies.”

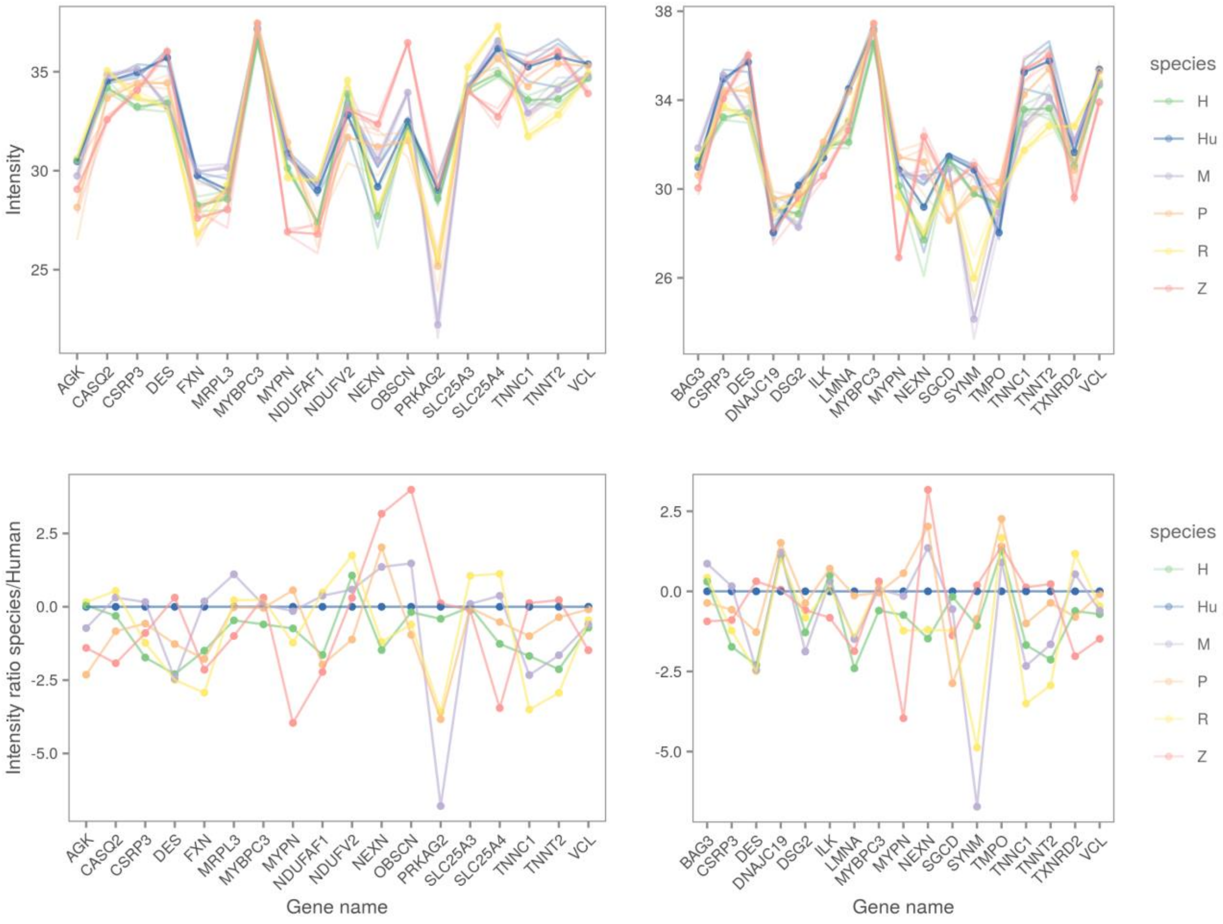

Figure S16: Profile plots of protein expression of disease-related genes in left ventricle of all species measured. Genes related to hypertrophic cardiomyopathy (HCM) are shown on the left, dilated cardiomyopathy (DCM) on the right. Top row: absolute protein intensity on log10-scale. Faint lines depict single measurements, points and thick lines show mean values. Bottom: Ratio of protein intensity of each species relative to human, calculated from mean protein intensities, separately for each protein. Species abbreviations: H - horse, Hu - human, M - mouse, P - pig, R - rat, Z - zebrafish. Only proteins with no missing data points were included.

7. The authors suggest that pigs make the best ARVC models based on the proteomics results, but mice are often used in ARVC studies. Can the data tell us anything on what might be the caveats there and how to potentially make new/better mouse ARVC models?

What our data suggest is that the protein abundance profiles of the proteins known to be involved in the disease do not have the same abundance profiles in mice as they do in the human heart. One may hypothesize that an improved mouse model of ARVC may be obtained by manipulation expression of the six ARVC genes in the right side of the heart exclusively.

However, such manipulations may have other unforeseen consequences, where it may be questioned whether performing the experiments in another animal is anyway more feasible.

8. The inverted atrium/ventricle distribution is a surprising and remarkable results. Are there orthogonal lines of evidence that can corroborate this finding. In the human dataset here, is there a strong agreement with the relative A-V distribution of proteins compared with previous datasets (Human protein atlas, Doll et al. Nat Comm 2017, etc.)

We agree with the reviewer on the importance of double-checking surprising findings against independent data sources, and have made an effort to validate our findings.

For human, we compared our chamber-specific human cardiac proteome data with two previously published studies that provide chamber-specific data at similar depth of proteome coverage (Linscheid et al., 2020 and Doll et al, 2017). To assess whether the relative A-V distributions showed agreement between studies, we plotted log2-differences in atrial vs. ventricular protein abundance from each study against our data and assessed how many of the proteins deemed significant in our study were found to have the same tissue enrichment in the other studies. Compared with our previous study employing similar methods and instrumentation (Linscheid et al., 2020), we found that 93% or 96% of proteins determined as significantly higher expressed in atria or ventricle, respectively, showed the same tissue enrichment between the two datasets. Similarly, we found that 96% or 93% of proteins determined as significantly higher expressed in either atria or ventricle in our study showed the same tissue enrichment in Doll et al (2017).

For mouse, to the best of our knowledge, no other chamber-specific proteomic dataset is published to date. We could thus not confirm the chamber-enriched protein expression detected in our study against another published dataset. We did however run an independent duplicate experiment with 3 additional mice during preparation of our manuscript, providing independent measurements of chamber-specific protein expression in the murine heart. This experiment was performed with the same protocol on the same instruments, but on different biological samples. To validate our findings in mouse heart, we here compared fold-change differences between atrial and ventricular protein expression between both datasets and found overall large agreement (Fig. S14). Note that the proteins from other studies were not necessarily deemed significant, so some deviations occur between studies. However, for the majority of proteins, the studies agree on the relative enrichment in either atria or ventricle.

We have added the following to our manuscript on page 7: “We confirmed the chamber-enriched expression of these proteins by comparison against independent datasets (Sup. Figure S14-15).”

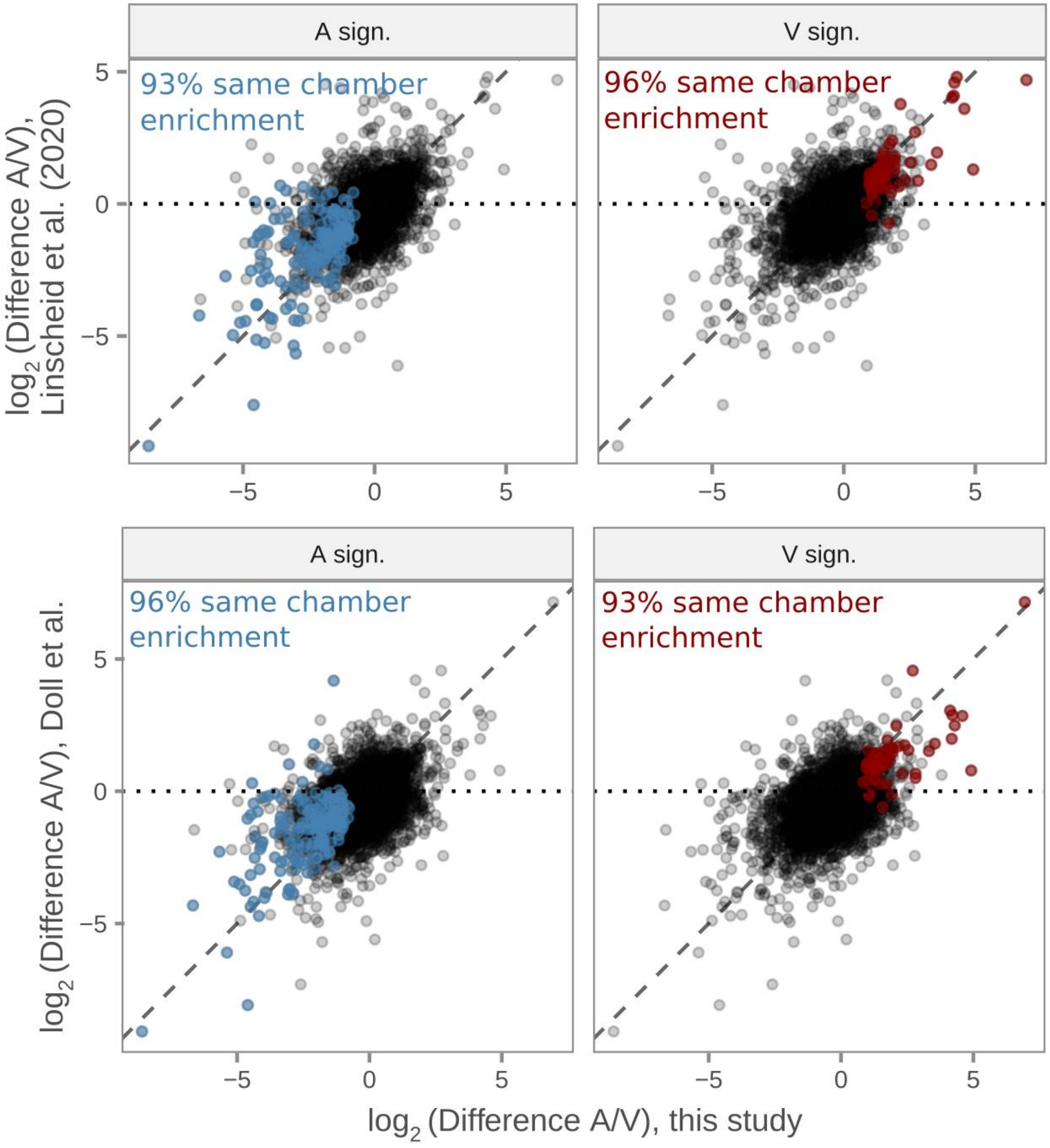

Sup. Figure 14: Comparison of protein expression differences between atria and ventricle with other studies. Protein abundance ratios between atria and ventricle as reported in this study (x-axis) were compared to two independent studies reporting chamber-specific protein expression in human hearts (y-axis). Top: Comparison to Linscheid et al. (2020), bottom: comparison to Doll et al. (2017). Proteins deemed significant in either arita (blue) or ventricle (red) in our study were highlighted and agreement on higher expression in the same chamber was determined for each study as annotated.

log2(fold-change, A/V)

Gene name	Human, this study	Human, Linscheid et al.	Human, Doll et al.	Mouse, this study	Mouse, replicate
DHRS7C	4.48	3.80	0.89	1.04	0.91
MTUS1	4.37	1.14	0.46	-0.46	-2.15
AOX1	4.15	2.61	2.95	-0.99	-1.08
PM20D2	3.34	0.32	2.67	-1.40	-0.74
CHL1	2.73	3.96	2.64	-1.00	1.82
MDN1	2.71		2.19		
KALRN	2.59		0.15	-1.14	0.11
BCHE	2.51	2.06	1.18	-0.54	-0.27
TP53I11	2.41	1.60	2.62	-0.87	0.00
RAB3IL1	2.09	0.21	-1.78	-1.36	-1.16
ANG	2.05	0.77	2.90	-0.53	0.00
DBN1	1.95	1.17	1.24	1.36	2.04
NES	1.84	1.44	1.11	-0.74	-0.10
RPS17	1.75	0.64	0.20	-0.32	-1.00
CYP27A1	1.74	1.59	1.83	-0.41	-1.31
CETN2	1.71	1.11	1.93	-0.80	-0.55
IWS1	1.61	-0.44	1.38	-0.65	0.07
RPS14	1.51	0.21	0.07	-0.32	-1.34
WIPF1	1.37	0.35	0.18	0.18	-0.48
RAI14	1.32	0.95	0.88	-0.40	0.49
SIRPA	1.16	-0.17	1.65	-0.36	-1.17
ACSF2	1.15	2.11	1.09	-2.37	-2.79
RPS25	1.11			-0.19	
RPL23A	1.05	-0.10	0.22	-0.39	-1.24
RELA	1.02	0.15	0.43	-0.64	-0.20
GRB2	0.83	1.31	0.54	0.35	-0.24
LSM6	0.80	-0.43	1.01	-0.05	0.50
EEFSEC	-0.99	-0.81	-0.40	0.12	1.47
CLNS1A	-1.07	-0.58	0.19	0.75	0.89
TPP1	-1.16	-1.02	-0.95	-0.15	-0.03
NPC2	-1.19	-0.12	-0.95	1.14	0.64
PPT1	-1.28	-0.67	-0.42	0.11	-0.89
DSP	-1.42	-1.39	-0.93		0.22
PLA2G15	-1.53	-1.95	-0.92	1.69	
BID	-1.63			0.58	
TIAL1	-1.72	0.72	-0.28	1.72	-1.04
EPDR1	-2.16	-3.78	-1.69	0.74	1.06
DDO	-2.55	-1.57	-1.56	-1.60	-0.68
LARP4B	-4.92	-1.30	-0.78	-0.02	-2.16

Sup. Figure 15: Fold-change protein intensity between atria and ventricle from this study compared to independent datasets. Fold-changes from Fig. 4d were compared with independent chamber-specific proteome datasets. Human protein expression fold-changes were compared against fold-changes derived from Doll et al. (2017) and Linscheid et al. (2020). Mouse data was compared against fold-changes in independent experimental data from 3 biological replicates (unpublished).

Minor Comments:

1. Study limitations should be moved from supplementary methods to main text.

We have the study limitations as a natural part of the Materials and Methods section, where it is presented just before the section on data availability. If journal style allows for having the entire methods section in the main manuscript, we would certainly find that an advantage.

2. Figure 5a/b heat maps -- very difficult to see which value is missing due to similarity of color for NA values with the color scale.

We acknowledge the point raised and we have accordingly modified the figure. Please see adjusted figure as outlined below:

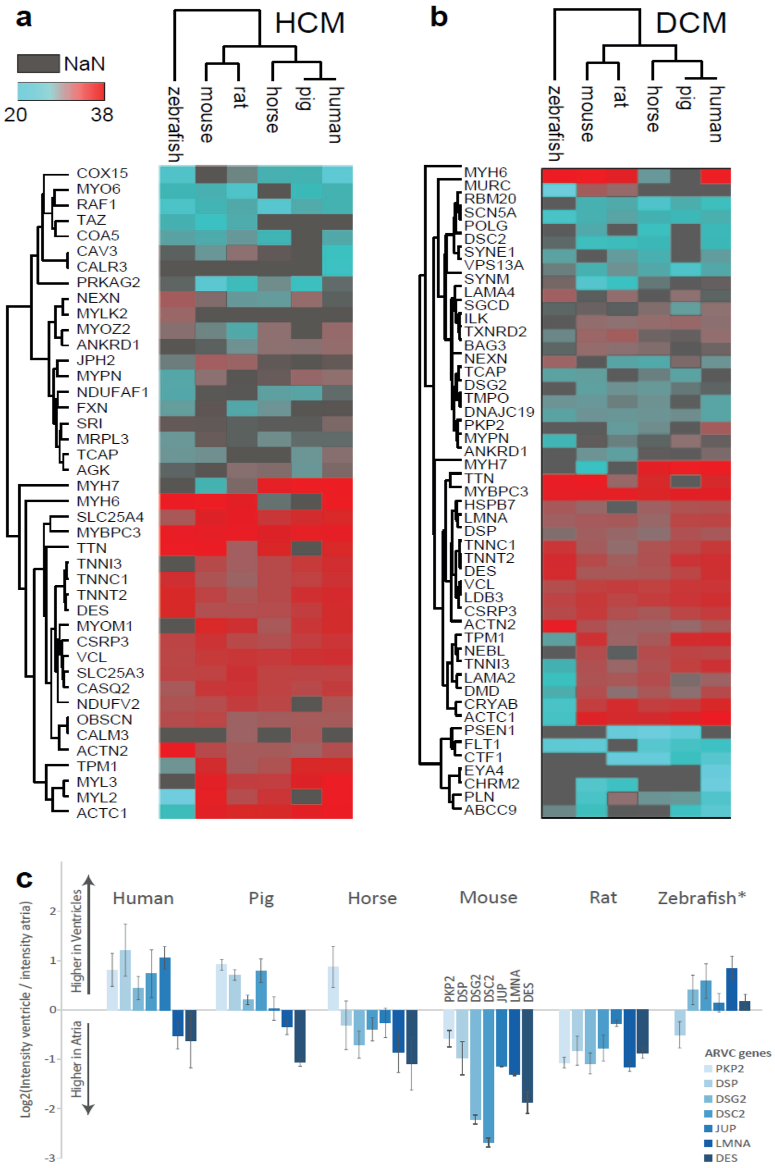

Supplement: Attachment — Submitted filename: Point-by-point Response to Reviewers on MS ID.pdf [file pbio.3001144.s027.pdf]
