# Supplementary material for: Quantitative proteome comparison of human hearts with those of model organisms
Source: PLoS Biol. 2021 Apr 19;19(4):e3001144. doi: 10.1371/journal.pbio.3001144 (PMC8084454; doi:10.1371/journal.pbio.3001144)
Supplement: Attachment — Submitted filename: Response to reviewers.docx [file pbio.3001144.s028.docx]

Rev. 2:

The authors have addressed most of the main concerns, and the revised paper is now suitable for publication.

We would like to thank the reviewer for a rigorous evaluation of our work, and for supporting publication of the study in *Plos Biology*.

Rev. 3:

Thank you for addressing my previous comments. I have no additional comments and look forward to seeing the manuscript published.

Thank you so much for the time and efforts invested in reviewing our work, and for supporting publication of the manuscript in *Plos Biology*.
